# Green Light Photoelectrocatalysis with Sulfur‐Doped Carbon Nitride: Using Triazole‐Purpald for Enhanced Benzylamine Oxidation and Oxygen Evolution Reactions

**DOI:** 10.1002/advs.202300099

**Published:** 2023-02-23

**Authors:** Maria Jerigova, Yevheniia Markushyna, Ivo F. Teixeira, Bolortuya Badamdorj, Mark Isaacs, Daniel Cruz, Iver Lauermann, Miguel Ángel Muñoz‐Márquez, Nadezda V. Tarakina, Nieves López‐Salas, Oleksandr Savateev, Pablo Jimenéz‐Calvo

**Affiliations:** ^1^ Department of Colloid Chemistry Max‐Planck‐Institute of Colloids and Interfaces Am Mühlenberg 1 14476 Potsdam Germany; ^2^ Department of Chemistry Federal University of São Carlos São Carlos SP 13565–905 Brazil; ^3^ HarwellXPS Research Complex at Harwell Rutherford Appleton Lab Didcot OX11 0FA UK; ^4^ Department of Chemistry University College London 20 Gower Street London WC1H 0AJ UK; ^5^ Department of Inorganic Chemistry Fritz‐Haber‐Institut der Max‐Planck‐Gesellschaft Faradayweg 4–6 14195 Berlin Germany; ^6^ Department PVcomB Helmholtz‐Zentrum Berlin für Materialien und Energie Schwarzschildstraße 3 12489 Berlin Germany; ^7^ Chemistry Division School of Science and Technology University of Camerino Via Madonna delle Carceri Italy; ^8^ Present address: Department of Chemistry Chair of Sustainable Materials Chemistry University of Paderborn Warburger Str. 100 33098 Paderborn Germany; ^9^ Present address: Department of Materials Science WW4‐LKO University of Erlangen‐Nuremberg Martensstraße 7 91058 Erlangen Germany

**Keywords:** benzylamine photooxidation, disordered carbon nitride, photoelectrocatalysts, purpald, sulfur doping

## Abstract

Materials dictate carbon neutral industrial chemical processes. Visible‐light photoelectrocatalysts from abundant resources will play a key role in exploiting solar irradiation. Anionic doping via pre‐organization of precursors and further co‐polymerization creates tuneable semiconductors. Triazole derivative‐purpald, an unexplored precursor with sulfur (S) container, combined in different initial ratios with melamine during one solid‐state polycondensation with two thermal steps yields hybrid S‐doped carbon nitrides (C_3_N_4_). The series of S‐doped/C_3_N_4_‐based materials show enhanced optical, electronic, structural, textural, and morphological properties and exhibit higher performance in organic benzylamine photooxidation, oxygen evolution, and similar energy storage (capacitor brief investigation). 50M‐50P exhibits the highest photooxidation conversion (84 ± 3%) of benzylamine to imine at 535 nm – green light for 48 h, due to a discrete shoulder (≈700) nm, high sulfur content, preservation of crystal size, new intraband energy states, structural defects by layer distortion, and 10–16 nm pores with arbitrary depth. This work innovates by studying the concomitant relationships between: 1) the precursor decomposition while C_3_N_4_ is formed, 2) the insertion of S impurities, 3) the S‐doped C_3_N_4_ property‐activity relationships, and 4) combinatorial surface, bulk, structural, optical, and electronic characterization analysis. This work contributes to the development of disordered long‐visible‐light photocatalysts for solar energy conversion and storage.

## Graphical abstract

This work contributes to the development of disordered photocatalysts with a long‐visible‐light range for solar energy conversion and storage. S‐doped/C_3_N_4_ materials are synthesized using one standard precursor, melamine, and a second novel and unexplored S‐containing molecule, purpald. The tunable hybrids exhibit high dispersion and homogeneity of S atoms within the final C_3_N_4_ matrix with a significant S bulk and surface content.

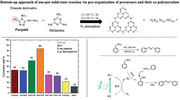

## 1 Introduction

Sustainable conversion of chemicals and green renewable energies are in high demand to meet proposed decarbonization targets.^[^ ^1^ ^]^ Existing industrial processes must be reinvented, and functional, stable, cheap, and abundant materials should be delivered to lower energetic and economical costs.^[^ ^2^ ^]^ Undoubtedly, the new generation of long‐range visible light high‐performing photoactive materials will play a key role in utilizing a significant portion of the solar spectrum while working under ambient conditions.^[^ ^3^ ^]^ The American Society of Materials^[^ ^4^ ^]^ points out that the visible light irradiating the Earth's surface is ≈43% of the total photons, thus representing the largest portion available (Air Mass 1.5 Spectra). The relevance of efficient visible light catalysts is driven by the urgency to exploit the sun's light (an abundant source).^[^ ^3^ ^]^

Photoelectrocatalysis is a leading field with the potential to unleash numerous noteworthy technological deliveries.^[^ ^5^ ^]^ Benchmark oxidative photocatalysis and photoelectrochemical cells (PEC) are of great interest to move forward the field in two directions: a) shifting conventional chemical processes and b) finding higher yields of solar‐to‐photon energy conversion for building block molecules or producing energy vectors. With respect to technological viability as a large‐scale implementation; one of the significant bottlenecks in the field is ensuring the materials in question are highly efficient light absorbers.^[^ ^6^ ^]^

Metal‐free, abundant, easy to modify, and active single‐catalyst are suitable candidates for diverse applications in the photo‐ and electro‐catalytic processes.^[^ ^6^ ^]^ A notable and simple route for the photoactivated production of the added‐value product is that of photocatalytic oxidation of benzylamine to dibenzylimine. Imines and their derivatives are important building blocks for the synthesis of several heterocyclic systems (imidazoles and palladacycles) and linear molecules (oximes and hydrazones) mainly used in the pharmaceutical and physiology industries with a market of ≈1 billion EUR and an annual growth of 11%.^[^ ^7^ ^]^ Though the conventional organic synthesis to obtain imines using metal salts based on aluminum, copper, nickel, or platinum results in high yields (80–95%),^[^ ^8^ ^]^ their protocols consist of thermal reflux and removal of water, making it costly and time‐consuming. Alternatively, photocatalysis can reach the same yields (≈90%) through one‐pot chemistry using simple control of atmosphere, substrate, light, and a “metal‐free” catalyst highlighting its wide‐scale feasibility by being a faster and greener process.

For photoelectrochemical oxygen evolution (OER), the reaction occurs in the anode of the electrolyzer. Yet, to this day, current state‐of‐the‐art of OER catalysts are based on metals such as iridium,^[^ ^9^, ^10^ ^]^ ruthenium,^[^ ^11^ ^]^ and iron,^[^ ^12^, ^13^ ^]^ from the oxides family, and others,^[^ ^14^, ^15^ ^]^ which are non‐abundant, have high/moderate‐cost, their raw materials availability may not secure large scale production demand, and dissolution problems.^[^ ^16^ ^]^ Thus, the replacement of traditional OER catalysts with organic semiconductors (SC) is an appealing strategy to mitigate the dependency on metal oxides.

Current metal‐free catalysts are graphene,^[^ ^17^ ^]^ covalent organic frameworks,^[^ ^18^ ^]^ carbon nanotubes,^[^ ^19^ ^]^ graphdiyne,^[^ ^20^ ^]^ and carbon nitrides (C_3_N_4_).^[^ ^21^ ^]^ Among them, C_3_N_4_ is an appealing SC due to its medium bandgap, for example, 2.7 eV that enables the capture of photons from 460 nm and below.^[^ ^22^ ^]^ Though activities are still rather low because of a) low absorption of photons in the visible range of the spectrum b) low charge carrier mobility, limited by the lack of interlayer hybridization c) its valence band position being too close to the O_2_/H_2_O potential, hindering water oxidation, d) high recombination, e) low surface area, etc.^[^ ^21^, ^23^ ^]^

Nanoarchitecture tailoring of C_3_N_4_ structural, electronic, and optical properties has been exhaustively investigated using four main strategies: templating, co‐polymerization, exfoliation, and elemental doping.^[^ ^24^, ^25^ ^]^ Such approaches aim at orienting planar bonding, symmetric interlayer interactions, control porosity, specific morphology, defined microstructure, and high surface area.^[^ ^24^, ^25^ ^]^

Elemental anionic doping is an established strategy to insert controlled atomic impurities to modify the C_3_N_4_ optical and electric properties.^[^ ^26^ ^]^ Among the possible anion dopant atoms: nitrogen, oxygen, boron, phosphorous, iodine, and fluorine, have proof of an effective but limited bandgap engineering reduction of the order of ≈0.2 eV.^[^ ^27^ ^]^ Although sulfur (S) doping provides an enhancement of light absorption of the same order, Density functional theory (DFT) has shown that the substitution of nitrogen atoms by sulfur dopants requires a low amount of energy, providing an upward shift of the conduction band, and a relative relax C_3_N_4_ matrix.^[^ ^28^, ^29^ ^]^ If the S‐atom is successfully inserted, it may serve as a multipurpose agent, such as: a) heteroatom active/trap site b) electron donor c) lattice distorter due to its large size d) polarizer to a lesser extent due to its p orbital disposition.^[^ ^23^, ^29^, ^30^, ^31^ ^]^

Despite the decade's progress of S‐doped/C_3_N_4_ the S‐containing most used precursors up to date have been: thiourea and trithiocyanuric acid. Such precursors allow a limited insertion of S atoms in the C_3_N_4_ lattice, exhibiting low S‐doped yields (up to 0.8 wt.%), owing to S moieties to leave upon calcination.^[^ ^23^, ^24^, ^32^, ^33^ ^]^ More recently, another S‐containing molecule were reported for similar reactions, the aminotriazoles but they exhibited a modest bandgap reduction.^[^ ^34^, ^35^, ^36^, ^37^ ^]^ There is an additional S‐doping strategy, using hydrogen sulfide (H_2_S) as a synthetic atmosphere, usually as a second post‐thermal treatment.^[^ ^32^, ^38^ ^]^ However, a predefined C_3_N_4_ structure should have been prepared in advance to permit the N or C subsequent substitution. Unlike typical and modern S precursors, purpald is an unexplored precursor containing an easy thiol‐leaving group that potentially facilitates the S atom insertion in the C_3_N_4_ chain.^[^ ^39^ ^]^

We report here, the rational design of highly dispersed and homogeneous S‐doped/C_3_N_4_ materials tested for benzylamine photooxidation (for the first time in literature, to the best of our knowledge), also for benchmark OER and, to a lesser extent, to capacitance performance. S‐doped/C_3_N_4_ materials were synthesized via a bottom‐up method via a one‐pot solid‐state reaction through the pre‐organization of precursors and their further co‐polymerization. One standard precursor, melamine, and a second novel and unexplored S‐containing molecule, purpald, were reported for the first time in literature. Both were used in different initial ratios to obtain five hybrid C_3_N_4_ materials with controlled S doping variation and two pure references. Interestingly, such tunable hybrids exhibited high dispersion and homogeneity of S atoms within the final C_3_N_4_ matrix with a significant S bulk and surface nominal content. Thanks to such tunability, the resulting S‐doped/C_3_N_4_‐based hybrid materials displayed improved optical, electronic, structural, textural, and morphological properties compared to the C_3_N_4_ references. In this study, we discussed, purpald as an S‐doping agent, how to add impurities in the C_3_N_4_ lattice to modulate its optoelectronic properties. This work differentiates from previous studies^[^ ^23^, ^30^, ^32^, ^33^, ^35^, ^36^, ^38^, ^39^, ^40^, ^41^, ^42^, ^43^ ^]^ thanks to the use of unexplored C_3_N_4_ precursor, simplicity (one‐pot reaction), effective (unusual higher) S atomic insertion by N substitution, and the resulting peculiar physico‐chemical features (describe in 2.5 correlations section), one can highlight the 1 eV optical bandgap reduction obtained.

## 2 Results and Discussion

### 2.1 Solid‐State Reaction Insights: Formation Path and Elimination Mechanism

To understand the formation of final hybrid carbon nitrides of this study, scientific criteria of the selected thermal steps of the reaction, pre‐organization of the precursor molecules, reaction mass yields, and TGA‐MS of the two precursors and the seven C_3_N_4_ materials were investigated. The nomenclature description of the C_3_N_4_ hybrid materials is based on the initial precursors’ ratio, for example, 10M‐90P, where M stands for melamine (10 wt. %) and P for purpald (90 wt. %). C_3_N_4_(P) is a calcined sample using purpald as the only precursor.

**Figure 1:**
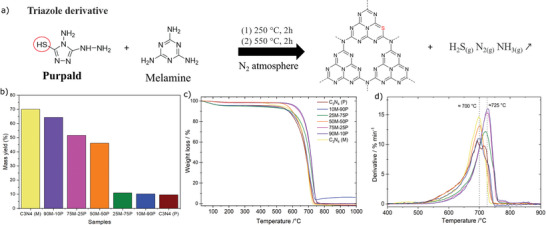
a) Synthetic path of the as‐prepared S‐doped/C_3_N_4_ with their elimination mechanism, b) Mass yield percentages, c) TGA profiles, and d) derivatives of all the series of the five hybrid materials and two references.

The one‐pot solid‐state reaction in **Figure** **1**a illustrates the combination of purpald and melamine precursors treated at two thermal steps under a nitrogen (N_2_) atmosphere, resulting in S‐doped/C_3_N_4_. The elimination mechanism is tentatively proposed as releasing the typical N_2_ and NH_3_ gases but also H_2_S due to the thiol group presence in the purpald molecule. Such a reaction aims to drive a pre‐orientation of the monomer precursors prior to the polycondensation. The two thermal steps serve different purposes: first, reaching 250 °C and keeping it constant for 2 h serves to solubilize purpald at 22 °C higher than its melting point (228 °C, Sigma Aldrich spreadsheet). This thermal step aims to form a liquid phase that entangles the melamine powder and creates an interface (liquid‐solid) contact and stimulates a closer molecular proximity between the two moieties (only valid for the hybrid materials). Second, reaching 550 °C and preserving it for 2 h serves to form the C_3_N_4_ heptazine polymeric chain, the well‐reported temperature in literature.^[^ ^44^, ^45^, ^46^, ^47^ ^]^

Thus, the proposed S‐doped/C_3_N_4_ molecule (Figure 1a), including only one S atom per C_3_N_4_ heptazine matrix is consistent with experimental and theoretical previous reports. The pioneering work on S‐doped C_3_N_4_ materials^[^ ^38^ ^]^ and a well‐supported mechanistic formation in S atoms insertion by substituting one N atom.^[^ ^33^ ^]^ The S‐doped/C_3_N_4_ molecule also respects DFT calculations^[^ ^29^ ^]^ and first principles,^[^ ^31^ ^]^ indicating that only one S atom per unit cell provides relaxed immediate atomic neighbor bonds. Importantly, more than one S‐inserted atom per unit cell will cause electronic charge redistribution and significant dissymmetry between the heptazine rings. Thus, our S‐doped/C_3_N_4_ molecule agrees with previously reported structures and theoretical conditions, respecting sulfur‐reported chemistry.

TGA profiles reveal the kinetic decomposition investigation of the purpald and melamine (precursors used). A brief study mimicking the solid‐state reaction shows the difference between the decomposition of melamine and purpald under an N_2_ atmosphere. The purpald decomposition occurs in three steps at 236, 511, and 665 °C with mass losses of 40, 5, and 55%, respectively as illustrated in Figure S1a (Supporting Information). As purpald is not listed among typical carbon nitride N‐rich precursors, we have tentatively attributed the three decomposition steps to the arrangement/formation of dicyandiamide (235 °C), passing by two intermediates melamine (340 °C) and melem (370 °C) to melon (510 °C) transition, and finally the 2D polymeric network of C_3_N_4_ heptazine (600–700 °C), in good agreement by experimental reports from both groups of Prof. Schnick^[^ ^45^ ^]^ and Prof. Lotsch based on variable temperature XRD.^[^ ^46^ ^]^ For the case of melamine decomposition, it occurs in a single step as illustrated in Figure S1b (Supporting Information), where all mass is lost at 340 °C consistent with the theoretical melting point occurring at 350–354 °C.^[^ ^48^ ^]^

The reaction mass yields were calculated when dividing the mass of the material recuperated after the thermal reaction in the crucible by the initial mass of the precursor(s) as noted in Equation (1).

(1) $$\%\;\mathrm{mass}\;\mathrm{yield}=\frac{m_{\mathrm{after}\;\mathrm{thermal}\;\mathrm{reaction}}}{m_{\mathrm{initial}\;\mathrm{precursor}(s)}}$$

The reaction mass yields of the resulting C_3_N_4_ (Figure 1b) provide a combination of a quasi‐linear descending trend (≤50% purpald) and a constant trend (≥75% purpald). For instance, the mass yield trend indicates two possible effects in the function of the varying precursor ratio: at high content of melamine (≥50%), the C_3_N_4_ preserves the quenching effect, and at low content of melamine (≤25%), or majority purpald content, the C_3_N_4_ hybrid materials experience an accelerated decomposition resulting in only 10% of the mass recovery.

TGA profiles of all the materials (Figure 1c) suggest that the thermal decomposition occurs in a one‐pot decomposition step, resulting in a quasi‐symmetrical peak in their respective derivatives Figure 1d. The decomposition starts progressively with a prolonged tail starting at ≈500 °C and finalizes with a pronounced decay at 775 °C, suggesting destabilization of the 2D structure materials begins gradually at lower temperatures before an abrupt decay above ≈700 °C. The temperature of the peak was observed at 700–725 °C, suggesting the presence of the interconnected heptazines (typical for polymeric C_3_N_4_), as has been previously reported in C_3_N_4_ studies.^[^ ^49^, ^50^ ^]^

Typical ammonia (NH_3_) and N_2_ gas traces from the C_3_N_4_ materials were monitored by TGA‐mass spectroscopy (Figure S2, Supporting Information). A third MS trace at m/z = 34 was monitored – ascribed to be H_2_S, produced from the thiol group of the purpald precursor.

Thanks to the H_2_S gas monitored (elimination mechanism), two hypotheses in the thiol chemistry functionality rose. Purpald thermal decomposition drives two benefits within this synthetic procedure: S‐doping in the C_3_N_4_ lattice and gaseous H_2_S release acting as a self‐porogen agent.

### 2.2 Physicochemical Characterization

To investigate and identify the dominant physicochemical properties of the resulting hybrid C_3_N_4_ in association with their performances a series of characterization techniques were performed. Optical and electronic properties were analyzed through a combinatorial approach of UV–vis–NIR, Photoluminescence (PL), and electrochemical Mott‐Schottky measurements.

**Figure 2:**
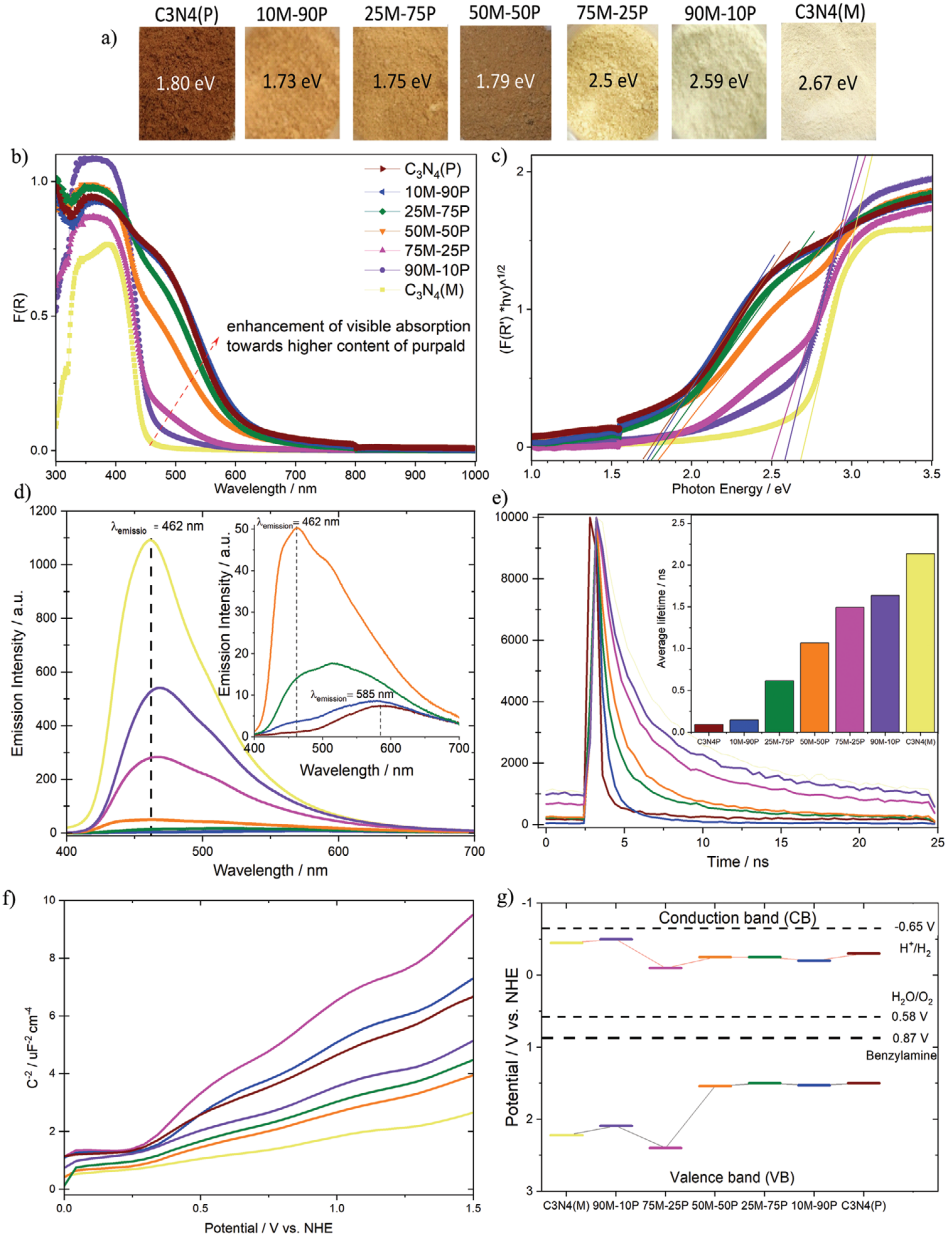
a) Color of the material powders of the five hybrid materials and two references with their optical bandgap (extracted from Tauc plot), b) UV–vis spectra, c) Tauc plot, d) steady‐state PL spectra (inset) zoom of the samples with high purpald content, e) transient PL spectra (Inset shows average lifetime quantification), f) Mott–Schottky plot g) flat band potential diagram of all the as‐prepared samples.

**Figure** **2**a shows the color of each as‐prepared sample. An observable trend from pale yellow (C_3_N_4_(M)) to reddish‐orange (C_3_N_4_(P)) is found. The two C_3_N_4_ hybrids with the highest content of melamine ≥75% remained in the yellow range while the other three C_3_N_4_ hybrids with high content of purpald ≥50% are orange/brownish, demonstrating that the mixing of melamine and purpald in different proportions results in tuneable optical properties. The UV–vis–NIR spectra (Figure 2b) reports the typical maximum absorption of C_3_N_4_ materials in the UV/blue range for all seven materials. Such a maximum is commonly associated with the absorption band edge at ≈455 nm, which in our study applies to all samples, particularly for C_3_N_4_(M), 90M‐10P, and 75M‐25P. This absorbance is ascribed to *π*→π* electron transitions of the aromatic *π*‐conjugated systems constituted of interconnected heptazine units.^[^ ^51^, ^52^, ^53^ ^]^ Although the two C_3_N_4_ hybrids with low purpald content (≤25%) exhibited a slight tail toward visible – samples with high purpald content (≥50%) transform the forementioned tail into a discrete shoulder toward higher wavelengths. This shoulder lacks a clear maximum but displays a red shift absorption band edge at 690–715 nm (representing ≈250 nm shift) equivalent to photon energies ranging from 1.7 to 1.8 eV. These photon energy values are consistent with the obtained optical bandgaps by the Tauc plot in Figure 2c and Table S1 (Supporting Information).

The observed tail/shoulder in the visible region may come from two associated contributions: a) S atoms substitution and b) *n*→π* electronic transitions. a) Due to the S larger size compared with C or N, S introduction in the C_3_N_4_ structure caused a significant lattice distortion^[^ ^31^ ^]^ and introduce a noticeable expansion effect or pronounced tilt angularity. Such distortion leads to new potential interactions in addition to Van der Waals, as an interplanar layer interaction/angularity is induced. A secondary reason should be considered to a lesser extent: the p*_x_* and p*_y_* orbitals of the introduced S atoms that partially donate electron density to the point of polarizing the s orbitals of the neighboring C or N atom, promoting new intraband energy states,^[^ ^31^ ^]^ despite the minimal electronegativity differences of S (2.5), C (2.5), and N (3.0) atoms.^[^ ^31^, ^54^ ^]^ The second potential contribution (b) has been attributed to *n*→π* forbidden electronic transitions, ascribed to a more pronounced layer deformation that results in a more distorted in‐plane configuration^[^ ^55^, ^56^, ^57^ ^]^ where electron lone pairs of the nitrogen edge atoms in the C_3_N_4_ skeleton are those responsible.^[^ ^39^ ^]^

In addition to these two explanations, secondary absorption bands at longer wavelengths have been recently studied for potassium poly heptazine imide (K‐PHI) systems, another member of the C_3_N_4_ family.^[^ ^58^, ^59^ ^]^ The noticeable shoulder was defined as the intraband states of this material that takes part in energy transfer reactions, typically with oxygen, under green or red irradiation. Therefore, we posit that similar behavior is expected for the C_3_N_4_ hybrids studied in this work. Overall, these new reddish orange materials (≥50% purpald) display an outstanding long‐range absorption in the visible range of the spectrum. This red‐shift enhancement of absorption is confirmed by color tuning from pale yellow to reddish‐orange and additional small intra‐band energy state signals.

Steady‐state PL spectra (Figure 2d) of the C_3_N_4_ present two different peaks and PL intensities vary among the samples underpinning their disparity in crystallinity. The first pronounced broad peak, centered at 462 nm was ascribed as the maximum emission for the most crystalline samples: C_3_N_4_(M) and the C_3_N_4_ hybrids with ≥50% of melamine. Subsequently, the PL intensity of the primary peak (462 nm) for the C_3_N_4_ purpald‐derivatives start decreasing when increasing the purpald content (≥25%) relative to the highest emission of the C_3_N_4_(M) (the most crystalline material). Nevertheless, the 50M‐50P C_3_N_4_ hybrids are the frontier at which high purpald content (>50%) C_3_N_4_ hybrids exhibit the second visible peak. Following the progressive apparition of the secondary peak centered at ≈585 nm (representing ≈123 nm shift), concomitant with a noticeable red shift, this suggests a more efficient photo‐induction of charge carriers separation occurring attributed to the reduction of charge density of the charge carrier traps, possibly by electron delocalization on surface terminal sites, evidencing how weak is the radiative recombination.^[^ ^32^, ^39^ ^]^ The red shift is particularly significant for the 10M‐90P hybrid and C_3_N_4_(P) reference, highlighting the enhancement of light absorption at longer wavelengths that may originate from the increase of structural defects, presumably by their disorder nature. These defects have been reported as new deep‐level traps for non‐radiative recombination, and electronic modulation properties in these new materials are expected.^[^ ^39^, ^60^ ^]^

Transient PL spectra (Figure 2e) display the lifetime of the charge carriers involved after the material was excited with a 375 nm energy pulse. A linear descending trend starting at the reference C_3_N_4_(M) exhibits the longest average lifetime charge carrier, 2.13 ns (Table S1, Supporting Information). The linear decay is followed by increasing the purpald content (and associated sulfur doping) in the hybrid materials, and the reference C_3_N_4_(P) exhibits the shortest average lifetime charge carrier, 0.09 ns.

Mott‐Schottky plots (Figure 2f) serve to determine the flat band potentials and reveal how the mixture of precursors affects the electronic band structures of the as‐prepared samples. The Mott–Schottky plots exhibited positive slopes, typically ascribed for n‐type SC.^[^ ^15^, ^39^, ^61^ ^]^ The flat band potentials were the conduction band (CB) position of each studied photoelectrode. In turn, the valence band (VB) positions were determined by subtracting the bandgap from the CB. The illustrative band energy potential diagram (Figure 2g) shows both CB and VB (Table S1, Supporting Information). The focus of this study is oxidation; thus, we will analyze the VB position in more detail. The VB values display a volcano plot trend with the most positive value at 2.4 V versus NHE for the 75M‐25P hybrid, and 0.3 V than its counterparts 90M‐10P and C_3_N_4_(M). The samples with ≥50% purpald exhibited very similar values, ≈1.5 V. Electronically speaking such results indicate that the 10–90% purpald content C_3_N_4_ hybrids follow the VB position of C_3_N_4_(P) whilst the high melamine content C_3_N_4_ hybrids an enhanced hybrid was formed, exhibiting an increased potential (+0.2).

Theoretical water (1.23 V)^[^ ^6^, ^62^ ^]^ and benzylamine (0.95 V)^[^ ^63^, ^64^ ^]^ oxidation potentials (selected model reactions) were added to the diagram for discussion. All samples in principle can thermodynamically perform both oxidations, regardless of the over potentials involved. One can presume that the 75M‐25P hybrid would undergo easier such oxidations due to its enhanced oxidation power, however, the pre‐requisite for undergoing oxidation is not limited to the VB edge position, since oxidation is a multi‐step process of photo (physical and chemical) steps involved. To contextualize the obtained values for C_3_N_4_(M) and C_3_N_4_(P) of similar materials, one can compare with mesoporous C_3_N_4_, Fe‐PHI, and K‐PHI giving 1.82,^[^ ^39^ ^]^ 2.70,^[^ ^65^ ^]^ and 2.54^[^ ^39^ ^]^ V versus NHE, providing consistency and reliability of the present M‐S measurements. Although the proton reduction is not at the core of this study, it can be said that according to the CB edges obtained (−0.1–−0.5 V vs. NHE), all the resulting materials can theoretically enable such reduction. Though the over potentials and energetic barriers for this reaction are more significant than for the oxygen half‐reaction and therefore, may be difficult to perform.^[^ ^66^ ^]^

**Figure 3:**
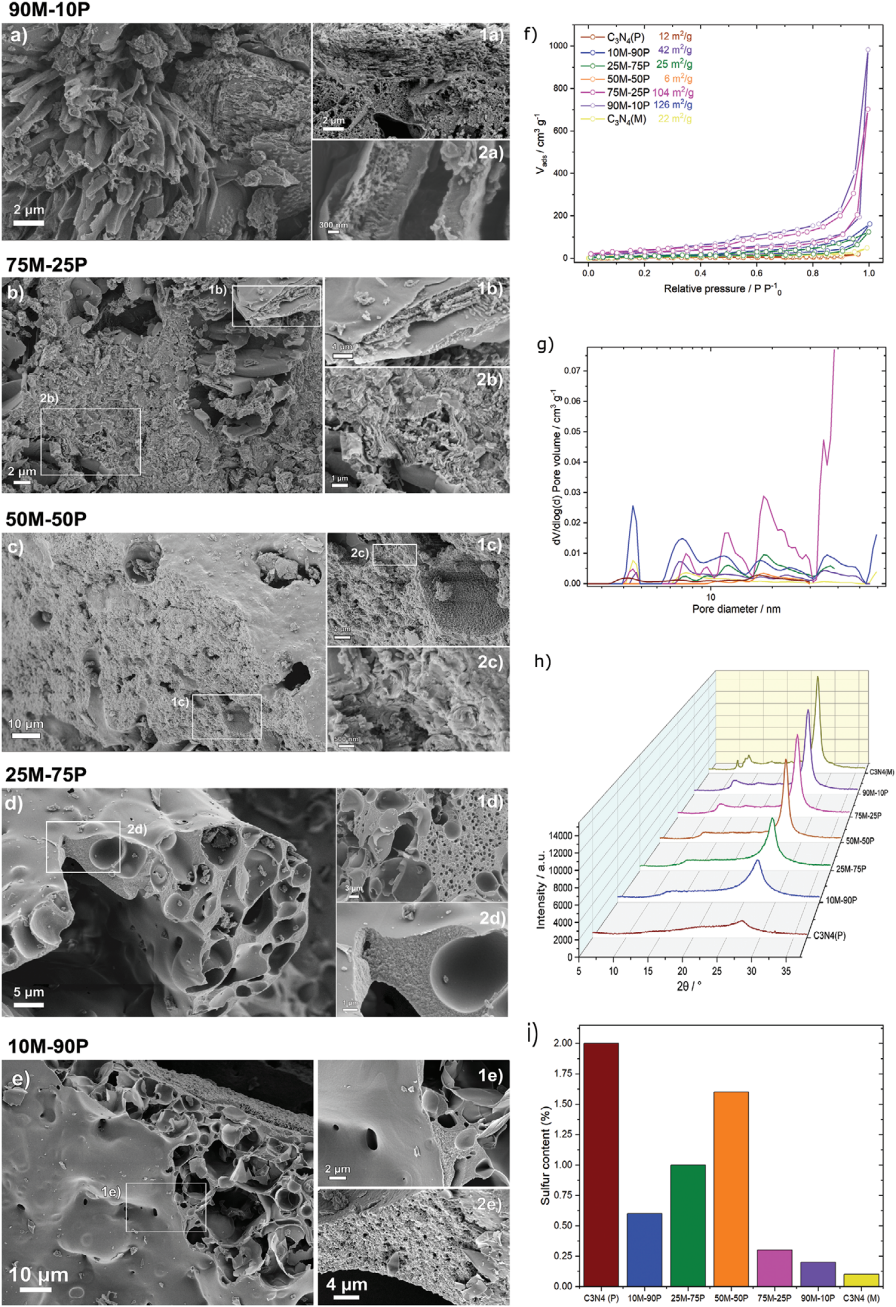
SEM images a) 90M‐10P, b) 75M‐25P, c) 50M‐50P, d) 25M‐75P, e) 10M‐90P f) BET specific surface area, g) pore size distribution, h) XRD patterns, and i) sulfur content of all the as‐prepared samples.

SEM images (**Figure** **3**a–e; Figure S3a–f, Supporting Information) reveal the morphologies of the five hybrid materials and C_3_N_4_(M & P). Figure S3a,b (Supporting Information) shows that C_3_N_4_(M) represents the morphology of the layered materials, with aligned platelets oriented differently at the surface and inside the particles. A deeper look into the C_3_N_4_(P) reference can be found in Figure S3c–f (Supporting Information). Grains possess large regions of a flat surface and regions with spheroidal shape with some degree of surface roughness. Intriguingly, these spheroidal structures are presented in two forms: closed and open (dozens of micrometers). The spheroids are hollow, implying the formation of gas bubbles, presumably due to the release of H_2_S gas (formed in autogenous condition) coming from the thiol functionality of the purpald molecule that can lead to a self‐porogen effect, in agreement with the proposed elimination mechanism.

The C_3_N_4_ hybrids show a clear trend in their morphology in regard to the reference materials' structure. 90M‐10P and 75M‐25P hybrids (Figure 3a,b) showed stacking of flat layers, similar to the C_3_N_4_(M) sample, however with considerably more disorder in between layers and a high number of pores. In addition, 90M‐10P reveals some hollow tube shapes (stalagmite‐like), with a potentially reasonable explanation of how the material releases gases formed during thermal treatment. 10M‐90P and 25M‐75P (Figure 3d,e) consist of grains with smooth surface layers under which two types of morphologies are hidden: spheroid pore shapes as we observed in C_3_N_4_(P) incorporated into more dense areas. The latter does not have any layered structure. In contrast, the 50M‐50P (Figure 3c) sample has the most unique morphology among all. It consists of large grains with a highly homogeneous smooth surface. The cross‐sectional view on the grains shows densely packed layers, which however all together appeared much wavier when in 25M‐75P. Large pores are present on the surface and the interior of the grains. Since the carbon nitrides references are morphologically very different, varying the purpald/melamine ratio leads to different combinations of these morphologies, with the 50M‐50P making balance synergy. The self‐porogen effect (bubble signature as spheroid pore form) was essentially pronounced for the high purpald content (≥75%) samples. This porogen effect may be explained under two premises: a) purpald liquid solubility at 250 °C, entangling the melamine surface, and b) H_2_S elimination, driving different synergic effects at microstructural and morphological levels.

N_2_ adsorption‐desorption isotherms at 77 K of all the samples in Figure 3f show a combination of type‐IV isotherm and type‐H4 hysteresis loop. According to the IUPAC, this is ascribed to the presence of mesopores and slit‐like pores in the samples.^[^ ^67^ ^]^ The Brunauer–Emmett–Teller (BET) theory was used to calculate the specific surface area (S_BET_) of the materials. All the samples show low S_BET_ due to the very low adsorption at low relative pressures, except for 90M‐10P and 75M‐25P that show ≈126 and 104 m^2^ g^−1^ (**Table** **1**). Quenched solid density functional theory (QSDFT) was used to obtain the pore size distribution of the samples (Figure 3g). All of them show multimodal mesoporous profiles with pores ranging from 2 to 50 nm. 90M‐10P stands out due to subtle differences in pore architecture – possessing the highest density of small‐diameter pores (≈3 nm). 50M‐50P exhibited three pores between 10–16 nm close to the surface, with arbitrary depth (very low pore volume). The not well‐defined pore size distribution of the samples is ascribed to the different mechanisms of C_3_N_4_ phase formation, resulting in a range of mesopores cavities and depth combinations.

**Table 1:** Specific surface area, pore size/volume, mean crystallite size, number of layers, the sulfur content in the bulk, and C/N ratio of the as‐prepared materials

Sample	S_BET_ [m^2^ g^−1^]	Crystallite [nm]	Layers^a)^ [N°]	2*θ* [°]	FWHM	S bulk [wt.%]
C_3_N_4_(P)	12 ± 1	1.8 ± 0.4	5 ± 1	27.20 ± 0.01	4.8 ± 0.6	2.0 ± 0.2
10M‐90P	42 ± 4	2.4 ± 0.4	7 ± 1	27.26 ± 0.01	3.6 ± 0.4	0.6 ± 0.1
25M‐75P	25 ± 3	3.5 ± 0.4	11 ± 1	27.27 ± 0.01	2.4 ± 0.2	1.0 ± 0.2
50M‐50P	6 ± 1	7.2 ± 0.4	21 ± 2	27.28 ± 0.01	1.2 ± 0.1	1.6 ± 0.2
75M‐25P	104 ± 10	6.2 ± 0.4	18 ± 2	27.21 ± 0.01	1.4 ± 0.1	0.3 ± 0.1
90M‐10P	126 ± 13	6.5 ± 0.4	19 ± 2	27.22 ± 0.01	1.3 ± 0.1	0.2 ± 0.1
C_3_N_4_(M)	22 ± 2	7.1 ± 0.4	21 ± 2	27.25 ± 0.01	1.2 ± 0.1	0.1 ± 0.1

^a)^ Calculated as described by Saner et al.^[^ ^71^ ^]^

The XRD patterns (Figure 3h) exhibited the typical diffraction peaks for C_3_N_4_ at 2*θ* = 12.9° and 27.2° for all the samples, corresponding to heptazine motif repetition and interlayer stacking of *π*‐conjugated aromatic systems, respectively.^[^ ^21^ ^]^ The latter is the 002 diffraction plane, characteristic of graphitic materials.^[^ ^22^, ^51^, ^68^ ^]^ A qualitative study of the crystal size and d_space_ was performed to further exploit XRD data by an understanding of the atomic layer disposition on the C_3_N_4_ in‐plane. Considering that the studied materials are heterogeneous, supported by SEM, this analysis is valid knowing that XRD is an average measurement of the heptazine unit repetition. Given those diffractogram positions and using peak fitting to obtain their Full Width Half Maximum (FWHM, Table 1, Figure S4, Supporting Information), the d_space_ values were calculated using the Bragg equation at ≈6.90 Å and ≈3.36 Å, compared with the theoretical d_space_ values of a perfect non‐doped C_3_N_4_ with 7.13 and 3.26 Å. The reported decreased spacing between the repetitive heptazine units plus concomitant increase to interlayer stacking may be related to the inclusion of S atoms within the C_3_N_4_‐skeleton causing first an expansion of the lattice and second some in‐plane tilt angularity, resulting in major disorder degree.^[^ ^69^ ^]^ The mean crystallite size (Table 1) was calculated using the Debye‐Scherrer relationship. The two largest crystallites were observed for the C_3_N_4_(M) (7.1 ± 0.4 nm) and unexpectedly for the 50M‐50P hybrid (7.2 ± 0.4 nm). Once again, a volcano trend was found for crystallite size, indicating that at a ratio of 50M‐50P, the system is best compromised to preserve crystal size as of the C_3_N_4_(M) reference. As a general rule, larger crystallites are found in hybrid materials with a high melamine proportion ≥75%. In turn, smaller crystallite sizes are found for lower melamine content ≤25%. The smallest crystallite size was obtained for C_3_N_4_(P) with 1.8 ± 0.4 nm, the most disordered sample. This result implies that melamine load increases the crystal size while conversely, purpald content tunes the amorphicity of the obtained C_3_N_4_. One cannot exclude that purpald presence in the liquid phase solubilization or pyrolysis somehow inhibits the crystal growth, as has been observed in other S‐doped/C_3_N_4_ materials.^[^ ^70^ ^]^ The number of layers (Table 1) in the stack followed the same volcano‐shape trend, with the largest number of layers obtained for the 50M‐50P hybrid (21 ± 1) plus C_3_N_4_(M, 21 ± 1) samples, and the fewest layers in C_3_N_4_(P) (5 ± 1), meaning that as the disordered character increases, the material seems to be more exfoliated.

Elemental analysis was performed to determine the total sulfur, nitrogen, carbon, and hydrogen content (Table S2, Supporting Information). The S content of the C_3_N_4_ hybrids again exhibited a volcano trend (Figure 3i), excluding the highest S sample content, approximately C_3_N_4_(P) with ≈2 wt. %. The second highest S content was found for the 50M‐50P hybrid (1.6 wt. %), seemingly the optimal precursor ratio in preserving the S within the C_3_N_4_ structural lattice. Such bulk S content respects the theoretical maximum amount of S (3.5%) that a C_3_N_4_ can host in a relaxed structure by first‐principles calculations considering one S atom per unit cell.^[^ ^31^ ^]^ Further increasing purpald content (≥75%) in the C_3_N_4_ hybrids, induces a decrease in S content ranging between 0.6 and 1% whilst the low purpald (≤25%) C_3_N_4_ hybrids and the C_3_N_4_(M) reference present a very low amount of S (≤0.3%), almost at trace scale.

Nitrogen content varies marginally by ±3.9%, implying a plausible substitution of N by S atoms. For nitrogen content purposes, the samples are divided into three groups. The first consists of low purpald content (≤25%) plus C_3_N_4_(M) giving 62.3% of N on average, then we separated the exceptional 50M‐50P hybrid (61.3% N), and finally grouped the high purpald content (≥75%) of 58.9% average N. The latter trend indicates that if the purpald (S‐container) content increases in the mix, the lower nitrogen content, indicating a clear atomic substitution. For carbon, changing synthetic parameters results in a less significant variation than for N values. However, the samples can nonetheless be divided into high, intermediate, and low content as follows: 0.25, 50–90, and 100% of purpald, respectively. One cannot omit the possibility of C substitution by S atoms, if this hypothesis is indeed accurate, it will be true to a lower extent compared to N substitution, supported by the EA results. The C/N ratio for all samples was ≈0.56, which is lower than the perfect C_3_N_4_ (0.64),^[^ ^21^, ^51^ ^]^ suggesting incomplete condensation presumably due to the first thermal step that quenches/accelerates the sublimation simultaneously. In the same incomplete condensation context, the H content remains constant (2%), proof of an equal quantity of terminal amines for all the samples.

The FT‐IR spectra (Figure S5, Supporting Information) of all samples exhibited the same characteristic peaks of C_3_N_4._^[^ ^50^ ^]^ The broad absorption band (2900–3350 cm^−1^) was assigned to the N–H stretching vibration of secondary (C—NH—C bridge bond along the repeating motif phases) and primary amines (the terminating edge layer, C—NH_2_).^[^ ^49^ ^]^ One can also attribute them, to a lesser extent, to the hydroxyl O–H stretching due to the adsorbed water species.^[^ ^72^ ^]^ The defined sharp peaks in the range 1620 to 1230 cm^−1^ were ascribed to the fingerprint of heptazine units. A detailed explanation of the attribution of the different peaks was reported by Jimenéz et al.^[^ ^50^ ^]^ Two additional sharp bands were present (800 and 890 cm^−1^) and attributed to N‐H deformation mode and heptazine ring breathing mode.^[^ ^49^ ^]^ In conclusion, all 7 samples showed the characteristic C_3_N_4_ peaks and are consistent with the literature.^[^ ^73^ ^]^

**Figure 4:**
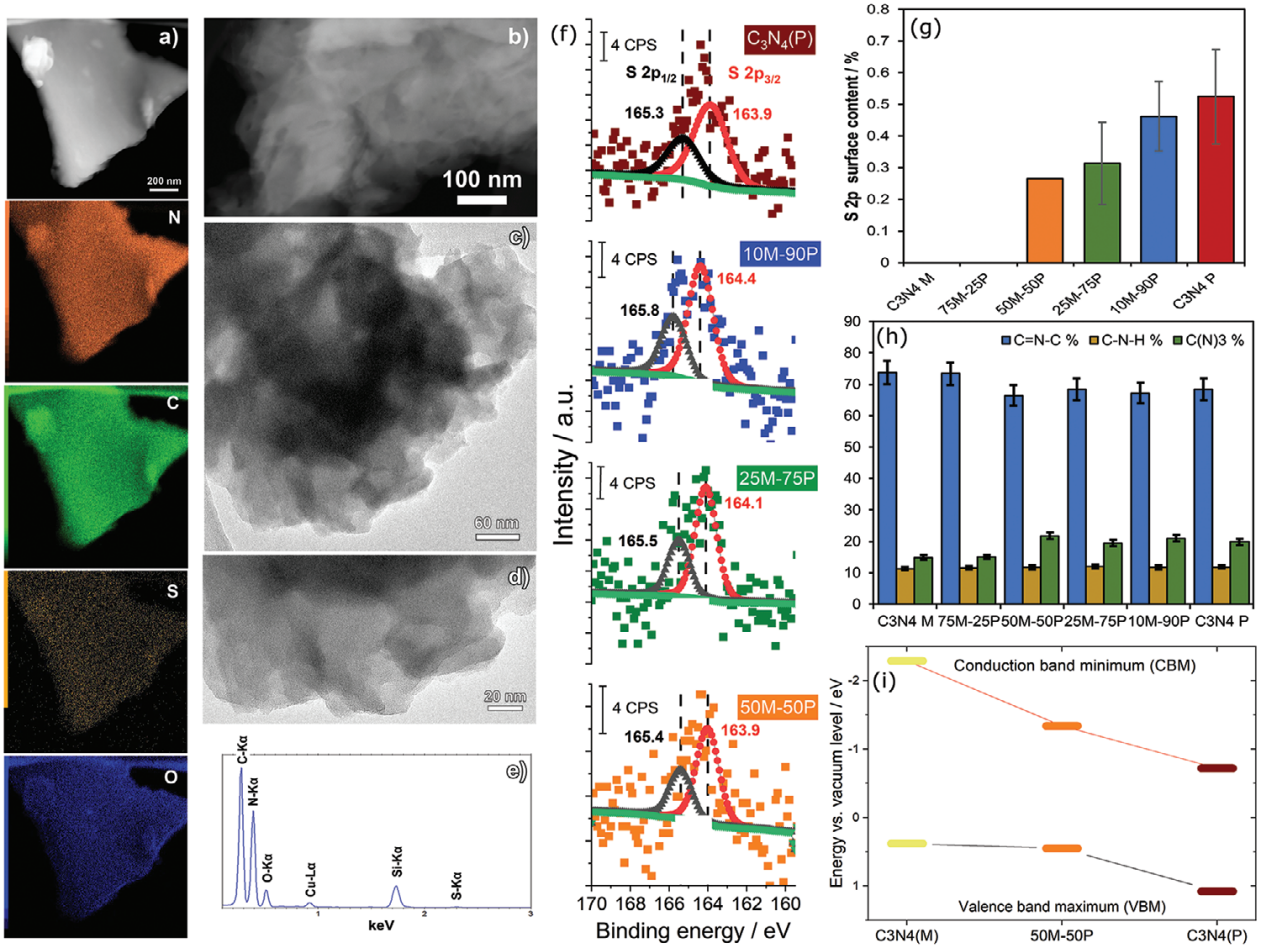
a) EDX element mapping distribution of 50M‐50P hybrid, b) ADF, c,d) HRTEM of 50M‐50P hybrid, e) EDX associated spectra, f) XPS S 2p spectra of the materials. Summary of the amount of g) sulfur (S2p XPS signal) and h) the different amounts of nitrogen functionalities (components of the deconvolution of the N1s XPS signal) obtained from XPS analysis. i) Energy band alignment diagram obtained from UPS results.

EDX elemental maps and the corresponding spectrum of the highest S‐doped (1.6 wt.%) 50M‐50P hybrid (**Figure** **4**a,e) confirmed the presence of N, C, S, and O atoms in the sample, homogeneously distributed on the surface. The lower presence of O can be explained through physically adsorbed O_2_ species and conjointly with SO_4_^−2^ detected after the thiol group of purpald decomposed.^[^ ^38^, ^74^ ^]^ The S content is the lowest signal as expected since it is in very low amounts (1.6 wt. % by EA). As suggested by DFT calculation, the S‐doping is preferentially oriented to substitute the sp^2^ nitrogen of the tri‐s‐triazine unit for relaxation aspects.^[^ ^29^ ^]^ The homogeneous S distribution along the grain surface points to its well‐distributed doping coverage. Interestingly, the same homogeneous distribution of S, N, C, and O was found for the C_3_N_4_(P) (Figures S6,S7, Supporting Information).

To compare materials at the nanoscale we performed high‐resolution transmission electron microscopy (HRTEM) and annular dark‐field scanning transmission electron microscopy (ADF) and bright‐field analysis of the two references and 50M‐50P hybrid. By ADF comparison (Figure 4b; Figure S8a,d, Supporting Information), a linear trend was found in brightness ranging from the brightest C_3_N_4_(P) toward the darker C_3_N_4_(M), due to the S content. HRTEM images (Figure 4c,d; Figure S8b,c,e,f, Supporting Information) revealed typical C_3_N_4_ lamellar texture, 2D interconnected layer morphology, and the edges appeared symmetrical to some extent. Interestingly, C_3_N_4_(P) material (Figure 4c,d), exhibits a more transparent domain, composed of a stack of well‐distributed layers and thus a thin or exfoliated stacked in agreement with XRD small crystallite size and a few layers. The 50M‐50P (Figure 4c,d) and C_3_N_4_(M, Figure S8d–f, Supporting Information) materials show a darker image contrast, potentially due to two effects: a) thicker stacks of layers compared to C_3_N_4_(P) and b) the layers are not homogeneously superposed between each other, given a non‐symmetrical entanglement.

Surface analysis of the obtained C_3_N_4_ materials was performed using X‐ray photoelectron spectroscopy, to elucidate the atomic bonding in the C_3_N_4_ skeleton and probe their local chemistry. XPS analysis will focus on the peaks of O 1s (532 eV), N 1s (400 eV), C 1s (285 eV), and S 2p (165 eV) that were fitted using modified Voight‐type line shapes. The high‐resolution XPS S 2p signal (Figure 4f) displays the single spin‐orbit doublet peak at ≈164 and 165 eV positions, ascribed as the S 2p_3/2_ and S 2p_1/2_ doublet of C‐S type sulfur, consistent with the literature.^[^ ^30^, ^31^, ^32^, ^33^, ^38^, ^42^, ^75^ ^]^ The 164 eV peak evidences the partial substitution of hybridized N by S atoms in the lattice.^[^ ^42^ ^]^ This finding is consistent with DFT calculations that revealed that the substitutional S atoms doping preferred to substitute the N over the C, for preservation in relaxed heptazine units.^[^ ^29^ ^]^ The 165,5 eV peak, besides the assigned C—S bond, can also be associated in lesser extent to low amounts of O_2_ absorbed species (S—O), consistent with Wang et al. finding.^[^ ^30^ ^]^

Figure S9a (Supporting Information) displays the deconvoluted N1s XPS spectra of the samples. The signals are deconvoluted in three well‐resolved peaks at 398.6 (most intense), 399.8, and 401.0 eV, ascribed as the sp^2^ hybridized pyridine N from heterocyclic aromatic rings (C=N—C bonds), tertiary bridges (N—C_3_), and amino groups (C—N—H).^[^ ^50^, ^62^, ^76^, ^77^, ^78^ ^]^ Figure S9b (Supporting Information) exhibits the classic three peaks of C at 284.8, 285.9, and the dominant peak at 288.3 eV. The 284.8 eV was assigned as adventitious carbon (C—C) or sp^3^ defects on the edge of graphitic domains or potentially as C—S bonds. Then 285.9 and 288.3 eV were assigned to the C—O as for adsorbed O_2_ species, supported by EDX, and sp^2^ coordinated N=C—(N)_2_ from the heterocyclic ring.^[^ ^50^, ^76^, ^77^, ^78^ ^]^ Interestingly, S 2p and N 1s signals of the C_3_N_4_ hybrids shifted slightly toward higher binding energies as purpald load increases, indicating that the S‐doping induced changes in the surface electronic C_3_N_4_ structure as previously seen by Wang et al.^[^ ^30^ ^]^ Figure S9c (Supporting Information) shows the O1s XPS spectra comprising two signals: 532,6 eV assigned to C‐O consistent with the 285.9 eV found from the C1s assignment and 531,5 eV attributed tentatively to the S—O bond of SO_4_^−2^ formation on the surface that was captured when the thiol group of purpald decomposes in H_2_S, as already proof by Liu et al.^[^ ^38^ ^]^

Regarding the quantitative XPS analysis of S 2p (Figure 4g). The S amount has a linear increase while S‐doping/purpald increases. Nevertheless, these surface quantities are 3–5‐fold lower than the bulk obtained by EA. This trend suggests that the incorporated S atoms in the C_3_N_4_ skeleton are partially embedded and partially on the surface. For the bulk S atoms may be accessible by the porous channels, whilst for the surface S atoms are exposed for immediate surface reactions. Figure 4h displays the sp^2^ pyridine N content remains constant until 25% purpald, and then decreases at contents of 50% and above, consistent with the increase of N atoms substitution by S. Since, the bridging tertiary N remains constant for the C_3_N_4_ hybrids (≥50%) and C_3_N_4_(P), no drastic changes in the interconnected heptazines may be inferred. However, a lower proportion of this tertiary N was found for C_3_N_4_(M) and 75M‐25P, presumably due to non‐symmetric atomic arrangements in the *x‐* or *y*‐axis of the heptazine direction. The amino groups are constant on the surface for all the samples in agreement with H content by EA.

Figure S9c (Supporting Information) shows the sp^2^ C of the 50M‐50P hybrid exhibits a higher content compared to its homologs, reinforcing the previously established hypothesis that the S substitution occurs mainly on the N rather than C. For the superposed C–S signal with adventitious C, 50M‐50P, and C_3_N_4_(P) have the highest value (20–24%), correlated to bulk S content. No differences were found for the C–O content among the samples, indicating that they probably adsorb the same quantity of O_2_ species.

The energy band alignment diagram (Figure 4i) was constructed from UPS results (Figure S10a, Supporting Information) and serves to determine how the S doping and defects contribute to the electronic modulation of the most S‐doped 50M‐50P hybrid relative to its two references. Theoretically, S impurities are considered anionic doping to substitute a majority of N atoms.^[^ ^27^ ^]^ The valence band maximum (VBM) of the 50M‐50P position is very similar to C_3_N_4_(M), emphasizing little change despite the 50‐50 initial mix of precursors, the hybrid preserves the electronic character of such reference. Nonetheless one cannot neglect the possibility of intraband states formation near the VBM, due to the upward increase compared to C_3_N_4_(P). Interestingly, the C_3_N_4_(P) has the most positive VBM of all (1.1 eV), suggesting a high capacity to undergo oxidations.

Looking at the conduction band minimum (CBM) of 50M‐50P is shifted toward positive and negative energy in reference to C_3_N_4_(P) and C_3_N_4_(M), giving a new intermediate hybrid CBM energy position. This new position may be explained by considering the synergistic effect of both precursors, and the successful S insertion, presumably promoting the S 2p orbitals interacting with the 2p of C from the *π*‐conjugated system or by the deep structural defects caused by the distorted lattice.^[^ ^31^, ^39^, ^60^ ^]^

Figure S10b (Supporting Information) displays the work function (WF) values of C_3_N_4_(M), C_3_N_4_(P), and 50M‐50P, calculated from a calibrated Fermi level with an internal gold reference. The resulting WF were 4.0, 3.1, and 3.2 eV, respectively. Considering that the theoretical WF of C_3_N_4_ is 4.65 eV,^[^ ^79^ ^]^ one can notice that the experimental value of C_3_N_4_(M) is similar. The difference between the other two samples, however, varies significantly (≈1.55 eV) potentially due to their disordered nature. The present solid‐state reaction comprehends two stationary temperatures, differing from the typical mesoporous or polymeric carbon nitride (single thermal treatment), modulating C_3_N_4_ structural, electronic, and optical properties, thus WF differences were expected.

### 2.3 Photooxidation Performance

**Figure 5:**
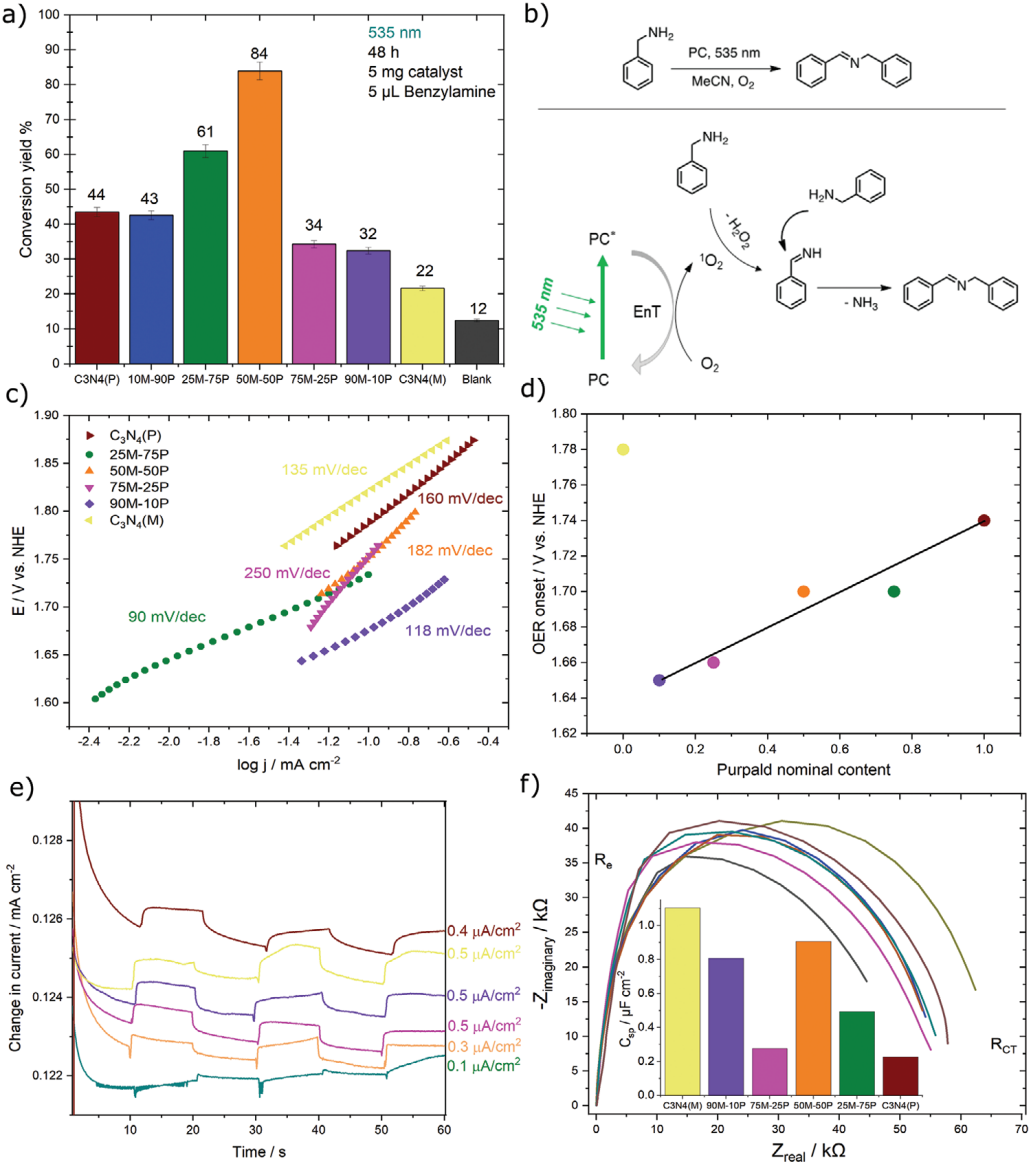
a) Photooxidation of benzylamine including the reaction conditions, b) illustrative mechanism and species involved in the photoconversion of benzylamine to dibenzylimine, c) Tafel slopes for OER reaction, d) purpald content in the function of OER onset values e) on‐off CA curves recorded using a white LED lamp, and f) Nyquist plot of all the materials.

The photooxidation conversion of benzylamine (substrate) to dibenzylimine was studied (**Figure** **5**a), upon monochromatic green light irradiation at 535 nm to excite (Emission spectra, Figure S11, Supporting Information) C_3_N_4_ materials, which is unusual, unless they have a great portion of visible light absorption. Figure 4 illustrates the volcano trend found for the series of the as‐prepared materials. Here, the 50M‐50P hybrid exhibited the highest conversion yield of 84 ± 3%, establishing it as the most active material of the series followed by 25M‐75P with a 61% conversion yield. The remaining samples have diminished conversion yields in the order of one, two, and threefold lower, for C_3_N_4_(P)/10M‐90P, 90M‐10P/75M‐25P, and C_3_N_4_(M), respectively. It is imperative to mention that a blank (experiment without catalyst) was conducted to account the contribution of photolysis (light) or thermolysis (temperature), determined to be 12% yield, almost half the capacity of the least active sample.

The mechanistic investigations of benzylamine oxidation were correlated with the electron paramagnetic resonance (EPR) technique. EPR tests were conducted to identify and detect the formation of singlet oxygen and or oxygen radical anion after an energy activation input at specific monochromatic light. Photocatalytic benzylamine oxidation has reported two possible mechanisms that may be also combined, namely electron and energy transfer, processes. First, electron transfer to the oxygen by forming an oxygen radical‐anion species, and second, through energy transfer to the oxygen, forming singlet oxygen.^[^ ^80^ ^]^ Both reactive species can take part in the subsequent oxidation of benzylamine. For that purpose, TEMP and DMPO were used to detect singlet and radical anion oxygen species, respectively, in dark and illuminate conditions. As the time irradiation used in the benzylamine photooxidation reaction was 2 days, we opt to use a stronger monochromatic light (higher energy input), 365 nm, for obtaining faster confirmation of oxygen species creation.

Figure S12 (Supporting Information) exhibits the radical scavenger EPR tests on the 50M‐50P hybrid material, the higher‐performing. Figure S12a (Supporting Information) confirms the formation of TEMP – ^1^O_2_ radical with a triplet signal under light irradiation as a result of energy transfer to oxygen and the formation of singlet oxygen. Similarly, Figure S12b (Supporting Information) represents a typical spectrum of DMPO – O_2_˙–, which confirms the O_2_˙– (oxygen radical anion) formation with quadruplet signal a quadruplet signal by electron transfer to oxygen. Thus, as hypothesized both oxygen species are formed at such experimental conditions. However, with respect to the previous studies,^[^ ^59^, ^81^ ^]^ we assume that the energy transfer mechanism is dominant upon 535 nm irradiation, as this low energy input was earlier shown to be not sufficient to induce electron transfer to O_2_. The narrow bandgap of 1.79 eV is sufficient in this term for photon absorption and energy transfer. The mechanism is sketched in Figure 4b.

### 2.4 Photoelectrochemical Performance

The oxygen evolution reaction (OER) was studied using the as‐prepared C_3_N_4_ materials as catalysts in alkaline media. Figure S13 (Supporting Information) shows the linear sweep voltammetry curves, exhibiting similar catalytic activity with close onset potentials. The onset potentials are plotted against the purpald nominal ratio in Figure 4d, showing that the C_3_N_4_ hybrids materials perform better than the two C_3_N_4_(M and P) references given their lower values (≤1.70 V vs. NHE). The hybrids’ onset potentials show a linear increase with increasing purpald content. Interestingly, the lowest onset is observed for the hybrid with only 10 wt. % of purpald. As the OER onset potential is highly influenced by the material conductivity. The best‐performing sample 90M‐10P has the highest surface area (126 m^2^ g^−1^), therefore its high performance is likely correlated to the accessibility to the catalytic active sites.^[^ ^82^ ^]^ The Tafel slopes (Figure 4c) exhibit a non‐linear trend as a function of the precursor nominal ratio. The photoresponse of the materials (Figure 4e) was measured at a bias of 0.6 V versus Ag/AgCl with a white LED lamp (Emission spectra, Figure S14, Supporting Information). The samples exhibit similar photoresponse except for 25M‐75P, showing negligible photoresponse probably due to fast charge carrier recombination. The observed samples' photoresponse are forward bias, typical for n‐type SC.^[^ ^16^ ^]^

The present C_3_N_4_ materials’ onset potentials are in the same range typically obtained for C_3_N_4_ with transition metals or hybrids C_3_N_4_ with heteroatom doping. Whilst the obtained Tafel slopes are high, the resulting materials exhibit slower performances than other reports, see Table S3 (Supporting Information). A previously reported S‐doped/C_3_N_4_ exhibited 1.42 V versus RHE onset, even lower than all the studied samples. However, its Tafel slope (120 mV dec^−1[^ ^75^ ^]^) is among the highest values in the literature and slightly lower that the ones reported in this study. We assume that the introduced sulfur atoms in the C_3_N_4_ skeleton result in a more hydrophobic surface. This hydrophobicity may hinder the proximity of reactants to the C_3_N_4_ vicinity, disabling effective oxidation of water by lowering the adsorption rate.^[^ ^83^ ^]^

Figure 4f displays the Nyquist plot and the values of specific capacitance. The electrode resistance is relatively low for all the samples, implying good interface contact of the material and electrode support. The charge transfer resistance of the C_3_N_4_ hybrids (≈60 kΩ) is lower than the two C_3_N_4_(M and P) references, though similar among themselves. The pure C_3_N_4_(M) shows the highest specific capacitance (1.1 µF cm^−2^). Surprisingly, the 50M‐50P sample has the second highest specific capacitance (0.9 µF cm^−2^) particularly close to the highest, although with fourfold lower surface area than C_3_N_4_(M). The high capacitance of C_3_N_4_(M) and 50M‐50P likely comes from the large crystal size, associated with the long atomic arrangement in the C_3_N_4_ skeleton that enhances the conductivity. Yet, the conductivity of an SC is not trivial to understand and may be influenced by many factors, for example, particle size, grain boundaries, crystallinity, heteroatom doping, and wettability, among others.^[^ ^84^ ^]^ Considering the specific capacitance differences of 90M‐10P and 50M‐50P compared to C_3_N_4_(P), this work implies that these hybrids may allow the new design of metal‐free capacitors by improving them with additional modifications via functionalization or vacancies creations, etc.

### 2.5 Structure‐Activity Correlations

Upon particularly low energy irradiation (535 nm – green light), the 50M‐50P hybrid material was the most photoactive catalyst in the benzylamine photooxidation reaction. In an attempt to link the resulting 50M‐50P sulfur doping tunability with the unique physico‐chemical effects obtained, we correlate the most pronounced properties with its photoactivity in this section.

The S‐doping of 50M‐50P hybrid material was evaluated in the bulk by EA (1.6 wt.%), in the surface by XPS (0.27%), and the elemental distribution by EDX (high dispersion and homogeneity), confirming the successful insertion of S atoms in the C_3_N_4_ lattice. Particularly, XPS provided the major evidence of S‐doping by the S—C bond presence, in agreement with DFT^[^ ^29^ ^]^ and first‐principles calculation^[^ ^31^ ^]^ that the S atomic insertion is preferentially substituting N atoms.

Such S‐doping insertion in the 50M‐50P structure resulted in several C_3_N_4_ modifications. For instance, the reduced optical bandgap (1.8 eV) with a conjoint absorption band edge red shift (≈250 nm shift) may be the most influential for photoelectrocatalysis. Thanks to the discrete shoulder covering a significant visible portion of the UV–vis spectrum compared to the classic C_3_N_4_ (M) material. This bandgap reduction is among the most pronounced in literature (generally ≈0.4 eV), to the best of our knowledge, and against its bare reference, for example, C_3_N_4_ (M). On top of the UV–vis findings, UPS, PL, and M‐S plots evidenced the formation of new intra‐band energy states by the new hybrid CBM energy position (middle value between the CBM of both references), two emission maxima (462 and 585 nm), and CB potential (1.5 V), allowing the oxidation of both target reactions: benzylamine and water, as theoretically and experimentally expected. Undoubtedly, the 1 eV BG reduction and new intra‐band energy states have exhaustively influenced photon absorption, capture, catalyst activation, creation, and concentration of excitons at the unconventional green light input.

The structural and morphological properties were also evaluated by XRD, BET, porosity, and SEM. 50M‐50P presented the largest crystallite size (7.2 ± 0.4 nm), a thicker stack of layers (21 ± 1), the lowest surface area (6 ± 1 m^2^ g^−1^), three surface exposed pores with arbitrary depth (10–16 nm), and a unique large grain with a homogeneous smooth surface. Those results emphasize the preservation of crystallinity despite the significant level of S‐doping. Additionally, a coexistence of macro and mesopores was observed by QSDFT and SEM microstructural features, which may facilitate the transfer of absorbed and desorbed moieties (a key step in electrocatalysis). Furthermore, the long atomic arrangement (good crystallinity) can be also beneficial for allowing the migration of charge carriers to undergo effective surface redox reactions.

In sum, a comprehensive characterization of the hybrid 50M‐50P was carried out. Its surface/structural new S active/trap sites in its C_3_N_4_ lattice resulted in light absorption enhancement and new intra‐band energy states. This enabled catalyst activation at the green light and transport phenomena improvements to form efficient singlet oxygen and oxygen radical‐anion (EPR) to perform the most efficient conversion of benzylamine to dibenzylimine (84 ± 3%).

## 3 Conclusion

A series of S‐doped/C_3_N_4_‐based materials were synthesized demonstrating enhanced optical, electronic, structural, textural, and morphological properties than their C_3_N_4_ references. The C_3_N_4_ hybrids exhibited higher performance in benzylamine photooxidation, oxygen evolution, and storing energy (capacitor brief investigation) than the references. Thus, this work serves to continue improving disordered catalysts with a long‐visible‐light range for solar energy conversion and storage.

The 50M‐50P hybrid exhibited the highest photooxidation organic conversion yield (84 ± 3%) of benzylamine to dibenzylimine in O_2_ atm upon green monochromatic light (535 nm) in 48 h. This result is attributed to the discrete shoulder in the visible light range (≈700 nm edge) due to the bandgap reduction of 1 eV of magnitude, the unusual high sulfur content (1.6 wt. %) for a carbonaceous material, the high dispersion and homogeneity of sulfur doping along its surface by substituting preferentially N atoms in the heterocycle, low porosity nature, new intraband energy states, preservation of crystal size, thick sheet stacking, rare deep structural defects due to layer distortion, flat surface morphology with a sharp roughness, and 10–16 nm pores.

## 4 Experimental Section

### Materials

4‐amino‐3‐hydrazino‐5‐mercapto‐1,2,4‐triazole or commercially called purpald (C_2_H_6_N_6_S, ≥99%, Sigma Aldrich), melamine (C_3_H_6_N_6_, 99%, Sigma Aldrich), ethanol (≥99%, Sigma Aldrich), potassium hydroxide (KOH, Alfa Aesar), Nafion (15–20% water, 1100 W, Sigma Aldrich), acetonitrile (CH_3_CN, Supelco, hypergrade for LC‐MS), benzylamine (99%, Sigma Aldrich) were used without further purification.

### Synthesis of C_3_N_4_ Materials

Graphitic carbon nitride references, were initiated with either melamine or purpald alone, and C_3_N_4_ hybrids were synthesized via thermal polycondensation under continuous N_2_ flow. The one‐pot solid‐state thermal polycondensation reaction was carried out in a static furnace applying a heating ramp of 1.9 °C min^−1^ until reached 250 °C and keeping it constant for 2 h and a second ramp of 1.7°C min^−1^ and keeping it constant at 550 for 2 h in a continuous N_2_ of 1 bar. For the C_3_N_4_ hybrids variation of the ratio of the two precursors (ranging from 10, 25, 50, to 75 wt. %) were put into an alumina crucible with a lid respecting the established mass ratio. For simplicity, the C_3_N_4_ hybrids were described with the initial mass ratio used, for example, 10M‐90P, where M stands for melamine (10 wt. %) and P for purpald (90 wt. %).

### Preparation of Electrodes

The working electrode's preparation consists of several steps. First, F‐doped tin oxide (FTO) glass was pretreated, cleaned by sonication in ethanol for 30 min and oven drying at 80 °C. The defined area of 0.25 cm^2^ of the FTO glass was protected using Scotch tape. Second, 3 mg of each material was weighed in a vial and 20 µL of Nafion (binder) and 0.2 mL of ethanol were added. This slurry was stirred overnight, then sonicated for 15 min, and 25 µL of the suspension was deposited by drop casting onto the pretreated FTO‐coated glass electrode (area 0.25 cm^2^). Finally, the electrode was air‐dried at room temperature for half a day, and the Scotch tape was removed and further dried at 120 °C for 2 h under a continuous flow of Argon to improve adhesion.

### Materials Characterization

Thermogravimetric analysis (TGA) was performed using a NETZSCH TG 209 F1 device. Each sample was placed into a platinum crucible and heated from room temperature (≈25 °C) up to 1000 °C with a heating rate of 10 °C min^−1^ under a nitrogen flow of 20 mL min^−1^. The standard deviation of the decomposition temperature of the analyzed samples was ±10 °C.

TGA‐MS measurement was performed using a thermo microbalance TG 209 F1 Libra (Netzsch, Selb, Germany) coupled with a Thermostar Mass spectrometer (Pfeiffer Vacuum; Asslar/Germany) with an ionization energy of 75 eV. A platinum crucible was used for the measurement of 10 ± 1 mg of samples in a Helium flow of 10 mL min^−1^ and a purge flow of 10 mL min^−1^. The samples were heated with a heat rate 2.5 K min^−1^ to 910 °C. Data were recorded and analyzed by the Proteus (6.1.0) and Quadstar‐software package (7.03, MID modus).

UV–vis–NIR absorption spectra were recorded on an Agilent Cary 5000 UV–vis–NIR spectrophotometer equipped with a diffuse reflectance‐integrating sphere. The acquiring starting and ending wavelengths were 300 until 1000 nm, with a scan time of 0.1, a data interval of 1, and a scan rate of 600 nm min^−1^. The bandgap was calculated by a statistically representative linear fit of the tangent decay curve (1–4 eV) by Tauc plot.

Steady‐state emission spectra (*λ*_ex_ = 365 nm) were recorded using an FLS980 photoluminescence spectrometer (Edinburgh Instruments Ltd, the United Kingdom) equipped with an ozone‐free Xenon arc lamp as the light source in a powder holder. Transient photoluminescence (*λ*_ex_ = 375 nm laser source) spectra were recorded using a PicoQuant FluoTime 250 fluorescence spectrometer equipped with PicoQuant PDL 820 picosecond diode laser controller. The experimental settings were: 10 000 counts, 25 ps resolution, 40 MHz frequency, 54.7° Polarizer, and 1 nm excitation bandwidth. The decay profiles were fitted using a multiexponential decay model and *τ* (average lifetime) of the charge carriers was reported following an existing methodology,^[^ ^85^ ^]^ fitting was done using Tau 2 software.

Elemental analysis (EA) by combustion was accomplished using a Vario Micro device.

Scanning electron microscopy measurements were performed using a Zeiss Leo 1550 equipped with a field emission gun and an Oxford Instruments energy‐dispersive X‐ray detector X‐MAX (80 mm^2^). Secondary electron images were recorded at 3 kV using an Everhart–Thornley detector.

Nitrogen adsorption/desorption isotherms were performed in a Quantachrome Quadrasorb SI‐MP porosimeter at 77.4 K. The samples were degassed prior to the measurements at 150 °C under vacuum (0.5 Torr) for 20 h in a 3 P Instruments Masterprep degassing machine. The Brunauer–Emmett–Teller (BET) model was applied to the adsorption isotherm to calculate the specific surface area from the data (0.05 < P/P_0_ < 0.2) using the QuadraWin 5.05. The total pore volume (VT) was calculated from the amount of gas adsorbed at P/P_0_ = 0.995. The pore size distribution was calculated by using a QSDFT model with a slit/cylindrical pore shape using the nitrogen adsorption data.

XRD measurements were carried out with a Rigaku Smart‐Lab X‐ray diffractometer equipped with a D/tex Ultra250 detector with Cu K alpha1 radiation (*λ* = 1.5418 Å) and Johansson monochromator. The scanning was performed at 2*θ* − *θ* mode, ranging from 5 to 40°, step size of 0.05, and speed of 2° min^−1^. Prior to scanning, the powders were inserted into an aluminum sample holder with a diameter and depth of 2.4 × 0.2 cm. Before each measurement, an optical and sample alignment were carried out to guarantee the best signal/noise ratio. The divergence slit was calculated through virtual crystallographic calculator V.2 from UCL, knowing the sample length (2.4 cm), lowest angle (5°) and the goniometer radius (30 cm). Beam stop and scattering slit plates were used to suppress the background and avoid scattering interference signals. Fitting the XRD patterns was performed through the Voigt method in Origin 2021b to calculate the FWHM and the position to apply the Bragg law and Debye‐Scherrer equations to get the d_space_ and crystallite size values of all the series.

Fourier transform infrared attenuated total reflectance spectra were recorded with a Thermo Fisher Nicolet iS5 spectrometer equipped with an attenuated total reflection unit of a diamond. Measurements were acquired in the 750–3700 cm^−1^ range with 32 scans and 8 cm^−1^ of resolution. The characteristic doublet of the atmospheric CO_2_ 2340/2360 cm^−1^ signal could not be removed from the spectra after background subtraction due to physisorbed CO_2_.

The Mott–Schottky measurements were performed in a Gamry Interface 1000 potentiostat using a 3‐electrode cell. The such cell consists of a Pt wire as a counter electrode, Ag/AgCl as a reference electrode, and FTO glass coated with the material as a working electrode. An aqueous solution of KOH (0.1 m) was used as an electrolyte and all the measurements were done at ambient temperature. The acquisition conditions of the samples comprised the potential range from −2 to 1.2 V RHE, 0.05 V potential step, and frequencies from 10 KHz to 100 Hz, 10 mV potential amplitude.

XPS data was acquired using a Kratos Axis SUPRA using monochromated Al K*α* (1486.69 eV) X‐rays at 15 mA emission and 12 kV HT (180 W) and a spot size/analysis area of 700 × 300 µm. The instrument was calibrated to gold metal Au 4f (83.95 eV) and the dispersion was adjusted to give a BE of 932.6 eV for the Cu 2p_3/2_ line of metallic copper. The Ag 3d_5/2_ line FWHM at 10 eV pass energy was 0.544 eV. The source resolution for monochromatic Al K*α* X‐rays is ≈0.3 eV. The instrumental resolution was determined to be 0.29 eV at 10 eV pass energy using the Fermi edge of the valence band for metallic silver. Resolution with the charge compensation system was <1.33 eV FWHM on PTFE. High‐resolution spectra were obtained using a pass energy of 20 eV, step size of 0.1 eV and sweep time of 60 s, resulting in a line width of 0.696 eV for Au 4f_7/2_. Survey spectra were obtained using a pass energy of 160 eV. Charge neutralization was achieved using an electron flood gun with filament current =  0.4 A, charge balance = 2 V, and filament bias = 4.2 V. Successful neutralization was judged by analyzing the C 1s region wherein a sharp peak with no lower BE structure was obtained. Spectra were charge corrected to the main line of the carbon 1s spectrum (adventitious carbon) set to 284.8 eV. All data was recorded at a base pressure of below 9 × 10^−9^ Torr and a room temperature of 294 K. Data was analyzed using CasaXPS v2.3.19PR1.0. Peaks were fit with a Shirley background prior to component analysis.

The UV source is a UVS 10/35 helium discharge lamp (SPECS), which supplied He(I) radiation @ 21.22 eV. For UPS, a system with a hemispherical electron analyzer (CLAM 4 by VG) and an electron detector of 9 discrete channeltrons was operated at 5 eV pass energy. The angle between the UV source and entrance cone of the analyzer is 54° with the electron lens placed perpendicular to the probed surface. A sputter‐clean Au foil served as a reference sample to determine the position of the Fermi edge. The sample was kept at −15 V versus ground to accelerate the emitted electrons toward the analyzer. For transmission electron microscopy (TEM) observations, a suspension of the sample in ethanol was sonicated for 10 min and then drop‐casted to the Cu grid with lacey carbon support and dried for 15 min. The TEM study was performed using a JEOL JEM‐F200 (S) TEM operated at 80 kV and equipped with a field emission gun and a high‐angle silicon drift Energy Dispersive X‐ray (EDX) detector (solid angle up to 0.98 steradians with a detection area of 100 mm^2^). TEM images were recorded using TVIPS TemCam‐F216. Annular Dark Field Scanning Transmission Electron Microscopy images were collected at a probe convergence semi‐angle of 25 mrad.

EPR measurements were conducted on a Bruker EMXnano benchtop X‐Band EPR spectrometer. The following settings were used for all spectra acquisition unless other is specified: Center Field 3444.05 G, Sweep Width 200 G, Receiver Gain 60 dB, Modulation Amplitude 1.000 G, Number of Scans 4, Microwave Attenuation 10 dB. Sample was placed and flame‐sealed in EPR capillaries (IntraMark, volume 50 µL, ID 0.86 mm), inside EPR tubes (ID 3 mm, OD 4 mm, length 250 mm). In situ EPR measurements of photocatalytic experiments were performed by coupling Thorlabs M415F3 Fiber‐Coupled LED (64 mW cm^−2^ measured at 0 cm distance) with Thorlabs DC2200 High‐Power LED controller.

### Photocatalytic TEMPO Detection Experiment

A solution of 2,2,6,6‐tetramethylpiperidine (TEMP) (5 µL, 0.03 mmol) in CH_3_CN (3 mL) was prepared in a 4 mL glass vial. The solution was flushed via the double needle technique with O_2_ for 2 min. The most active catalyst (50M‐50P) was prepared under the same reaction conditions, and it was introduced into a capillary (IntraMark, volume 50 µL, purchased from BRAND GMBH + CO KG) was sealed in the flame of the gas burner from one side. The capillary was charged with an aliquot of the TEMP solution in CH_3_CN (20 µL, 0.01 mol L^−1^). The open end of the capillary was sealed in the flame of a gas burner and placed into an EPR tube (ID 3 mm, OD 4 mm, length 250 mm). The EPR spectrum was acquired and used as a reference (0 min, in dark). Afterward, the sample was directly irradiated using a 365 nm LED module equipped with optic fiber, to perform in situ measurements. EPR spectra were acquired immediately when turning the light ON and after 30 min. The acquired spectra were compared with spectra obtained under the same conditions, but using clean uncoated glass capillaries (control experiments).^[^ ^81^ ^]^

### Photocatalytic DMPO‐O_2_•― Detection Experiment

A solution of 5,5‐Dimethyl‐1‐pyrroline N‐oxide (DMPO) (5 µg, 0.04 mmol) in CH_3_CN (3 mL) was prepared in a 4 mL glass vial. The solution was flushed via the double needle technique with O_2_ for 2 min. The most active catalyst (50M‐50P) was prepared under the same reaction conditions, and it was introduced into a capillary (IntraMark, volume 50 µL, purchased from BRAND GMBH + CO KG) that was sealed in the flame of a gas burner from one side. The capillary was charged with an aliquot of DMPO solution in CH_3_CN (20 µL, 0.01 mol L^−1^). The open end of the capillary was sealed in the flame of a gas burner and placed into an EPR tube (ID 3 mm, OD 4 mm, length 250 mm). The EPR spectrum was acquired and used as a reference (0 min, in dark). Afterward, the sample was directly irradiated using a 365 nm LED module equipped with optic fiber, to perform in situ measurements. EPR spectra were acquired immediately after turning the light source ON and after 30 min. The acquired spectra were compared with the spectra obtained under the same conditions, but using clean uncoated glass capillaries (control experiments).^[^ ^81^ ^]^

Irradiance of the LED modules was measured using PM400 Optical Power and Energy Meter equipped with the integrating sphere S142C and purchased from Thorlabs.

Emission spectra of LED modules were measured using Avantes spectrometer Avaspec‐ULS2048CL‐EVO‐R5 coupled with Thorlabs optical fiber M113L01 – Ø400 µm.

### Performance Tests

Photooxidation of benzylamine: In a vial of 4 mL, 5 mg of catalyst was weighed, and 5 µL of benzylamine and 1 mL of acetonitrile were added. The vial was saturated with an O_2_ atmosphere for 2 min. The vial was stirred at 800 rpm and exposed to a monochromatic LED green lamp light (535 nm, 50 W) for 48 h. After the reaction, a few µL were taken to fill the third part of a gas chromatography (GC) vial and completed with acetonitrile to quantify the resulting conversion to dibenzylimine via GC. The unconverted benzylamine was also quantified for internal control, both against a calibration curve using a standard dibenzylimine solution in Figure S15 (Supporting Information).

Electrochemical Oxygen Evolution: The electrochemical measurements, cyclic/linear sweep voltammetry (CV/LSV), and chronoamperometry (CA), were performed using the same 3 electrode cell as for the Mott‐Schottky experiments. LSV and CV curves were acquired at 10 and 30 mV s^−1^ of scan rate, respectively. The electrode potentials in all curves were converted to the reversible hydrogen electrode (RHE) scale using the following equation: E(RHE) = E(Ag/AgCl) + 0.197 + 0.059 × pH V. The onset of the OER was chosen as a potential needed to reach a current density of 0.25 mA cm^−2^.

Electrochemical Impedance Spectroscopy: Nyquist plots were obtained by measuring the galvanic electrochemical impedance spectroscopy (EIS). EC‐Lab software was used to extract from the Nyquist plot the two resistances (electronic and charge transfer) and capacitance values by using the circuit R1/(R2 + C2) configuration.

Photoelectrochemical Test: For photoelectrochemical measurements, the same electrochemical (3 electrodes) cell was used but with a white LED lamp (550.3 mW cm^−2^ at 8 cm distance, 50 W) at 4 cm distance from the working electrode to assess the photo response of the as‐prepared materials. The emission spectra are in Figure S9 (Supporting Information).

## Conflict of Interest

The authors declare no conflict of interest.

## Supporting information

Supporting Information

## Data Availability

The data that support the findings of this study are available in the supplementary material of this article.
