# Supplementary material for: Green Light Photoelectrocatalysis with Sulfur‐Doped Carbon Nitride: Using Triazole‐Purpald for Enhanced Benzylamine Oxidation and Oxygen Evolution Reactions
Source: Adv Sci (Weinh). 2023 Feb 23;10(13):2300099. doi: 10.1002/advs.202300099 (PMC10161101; doi:10.1002/advs.202300099)
Supplement: Supplementary file 1 — Supporting Information [file ADVS-10-2300099-s001.pdf]

ADVANCED SCIENCE
Open Access

Supporting Information

for *Adv. Sci.*, DOI 10.1002/advs.202300099

Green Light Photoelectrocatalysis with Sulfur-Doped Carbon Nitride: Using Triazole-Purpald for Enhanced Benzylamine Oxidation and Oxygen Evolution Reactions

*Maria Jerigova, Yevheniia Markushyna, Ivo F. Teixeira, Bolortuya Badamdorj, Mark Isaacs, Daniel Cruz, Iver Lauermann, Miguel Ángel Muñoz-Márquez, Nadezda V. Tarakina, Nieves López-Salas, Oleksandr Savateev and Pablo Jimenéz-Calvo**

Green Light Photoelectrocatalysis with Sulfur-Doped Carbon Nitride: Using Triazole-Purpald for Enhanced Benzylamine Oxidation and Oxygen Evolution Reactions

Maria Jerigova [1¥], Yevheniia Markushyna [1¥], Ivo F. Teixeira [1,2], Bolortuya Badamdorj [1], Mark Isaacs [3,4], Daniel Cruz [5], Iver Lauermann [6], Miguel Ángel Muñoz-Márquez [7], Nadezda V. Tarakina [1], Nieves López-Salas [1], Oleksandr Savateev [1], Pablo Jimenéz-Calvo [1 Δ, *]

*Corresponding author: pablo.jimenez-calvo@mpikg.mpg.de / pablo.jimenez.calvo@fau.de

[¥] Equal contribution of co-authors

[1] Department of Colloid Chemistry, Max-Planck-Institute of Colloids and Interfaces, Am Mühlenberg 1, 14476 Potsdam, Germany

[2] Department of Chemistry, Federal University of São Carlos, 13565-905, São Carlos, SP, Brazil

[3] HarwellXPS, Research Complex at Harwell, Rutherford Appleton Lab, Didcot OX11 0FA, United Kingdom

[4] Department of Chemistry, University College London, 20 Gower Street, London, WC1H 0AJ, United Kingdom

[5] Department of Inorganic Chemistry, Fritz-Haber-Institut der Max-Planck-Gesellschaft, Faradayweg 4–6, 14195 Berlin, Germany

[6] Helmholtz-Zentrum Berlin für Materialien und Energie, Department PVcomB, Schwarzschildstraße 3, 12489 Berlin, Germany

[7] Chemistry Division, School of Science and Technology, University of Camerino, Via Madonna delle Carceri, Italy

[Δ] Department of Materials Science WW4-LKO, University of Erlangen-Nuremberg, Martensstraße 7, 91058 Erlangen, Germany (current address)

List of contents

Figure S1. TGA decomposition profiles of purpald and melamine precursors.

Figure S2. TGA and MS ion current curves for the selected mass numbers measured for C_3N_4(P).

Figure S3. SEM images of a) C_3N_4(M) and b-e) C_3N_4(P) references.

Figure S4. Fitting of the XRD patterns of all samples.

Figure S5. FTIR-ATR spectra of the synthesized materials.

Figure S6. EDS mapping analysis of C_3N_4(P) reference.

Figure S7. EDS spectra of C_3N_4(P) reference.

Figure S8. Bright/dark field and HRTEM images of 50M-50P hybrid and two references.

Figure S9. XPS C and N spectra of four hybrids and two references.

Figure S10. UPS spectra of 50M-50P hybrid and two references including the fitting.

Figure S11. Emission spectra of the LED Green lamp, λ_{535nm} used for photooxidation measurements.

Figure S12. EPR spectra upon 365 nm irradiation and in dark of C_3N_4 50M-50P in a) DMPO and b) TEMP.

Figure S13. Emission spectra of the LED White lamp, λ_{365nm} used for PEC measurements.

Figure S14. LSV curves in dark conditions.

Figure S15. Gas chromatography calibration curve using dibenzylimine standard (AS1152B).

Table S1. Optical and electronic properties of the as-prepared C_3N_4 materials.

Table S2. Elemental analysis results of the as-prepared C_3N_4 materials.

Table S3. State-of-the-art photoelectrocatalyst with OER indicators to contextualize this study.

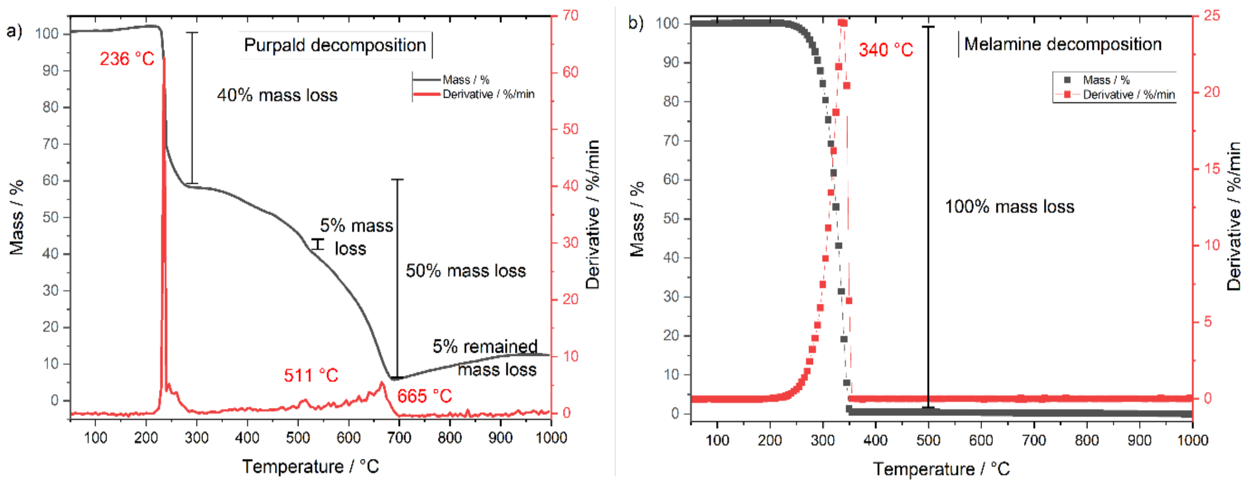

Figure S1. TGA decomposition profiles of purpald and melamine precursors under N_2 atmosphere.

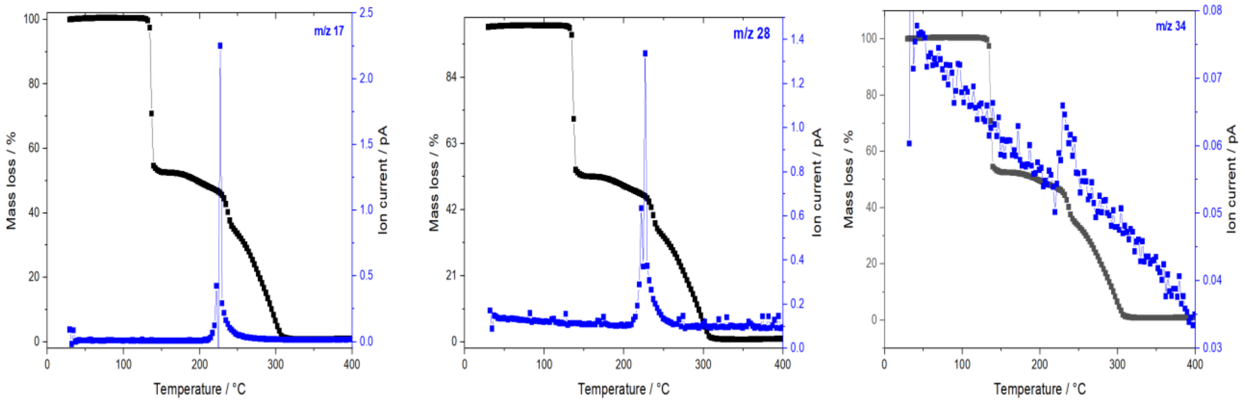

Figure S2. TGA-MS ion current curves of the purpald precursor.

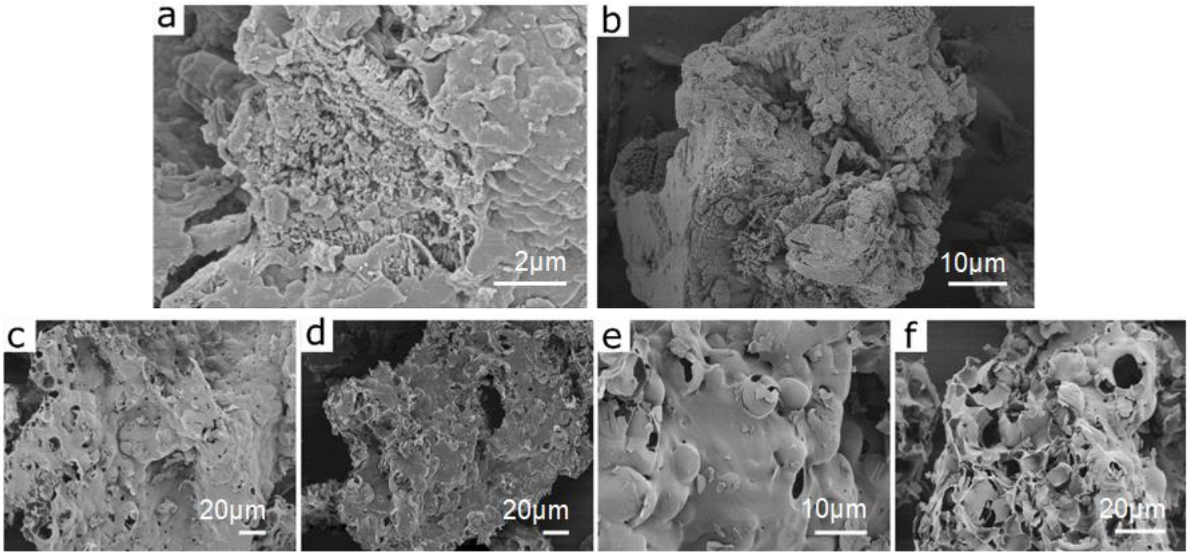

Figure S3. SEM images of a-b) C_3N_4(M) and c-f) C_3N_4(P) references.

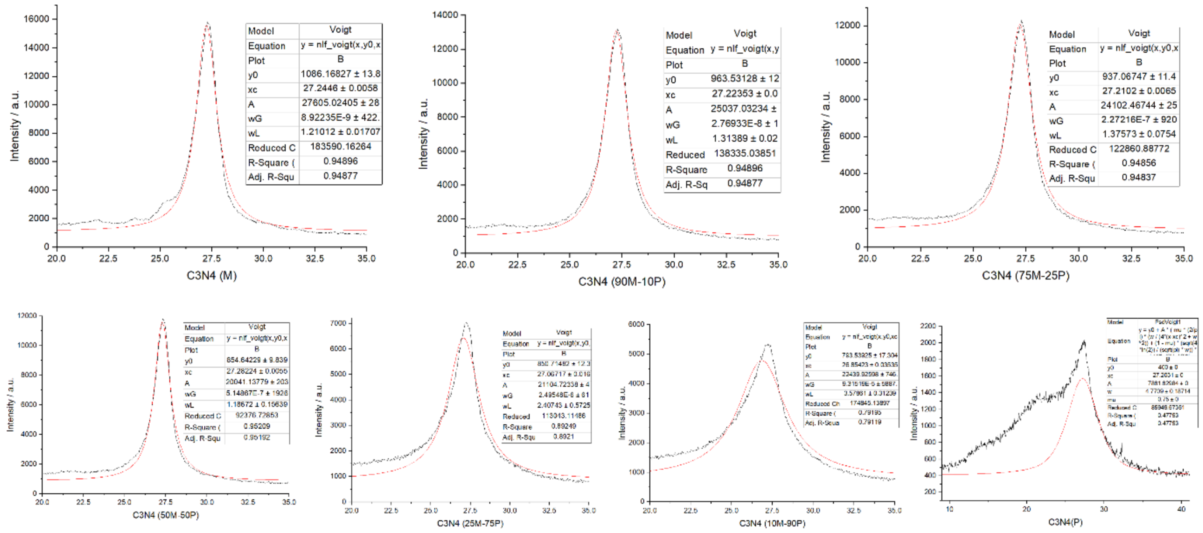

Figure S4. Fitting of the XRD patterns of all the samples.

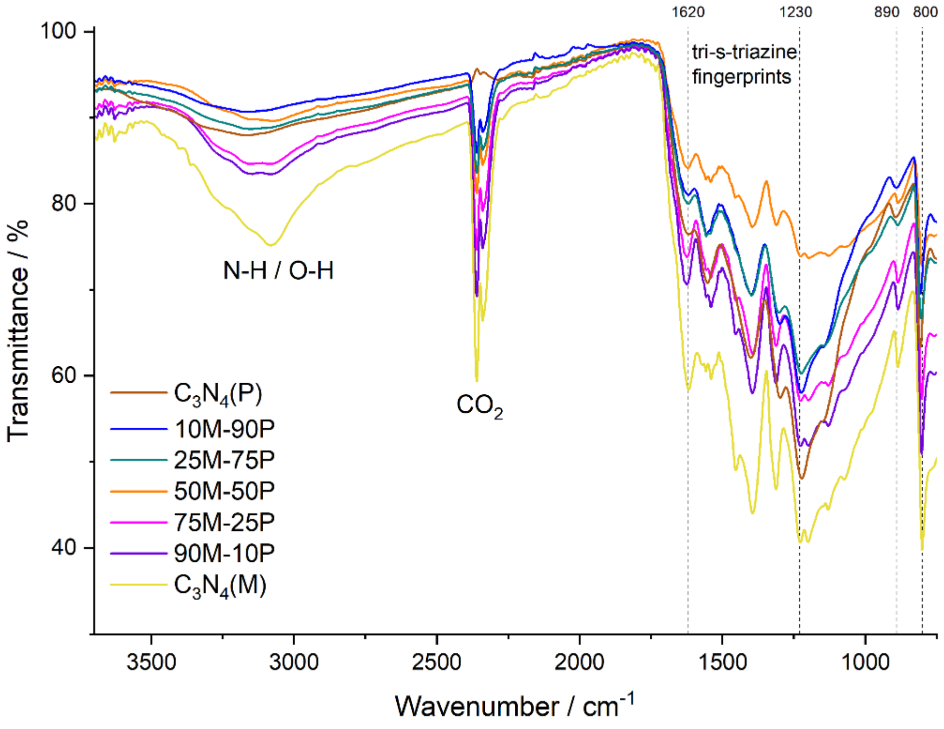

Figure S5. FTIR-ATR spectra of all the as-prepared samples.

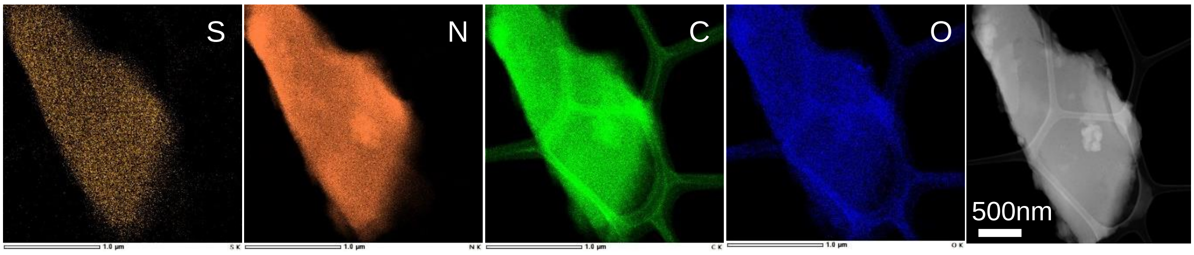

Figure S6. EDS elemental mapping of C_3N_4(P).

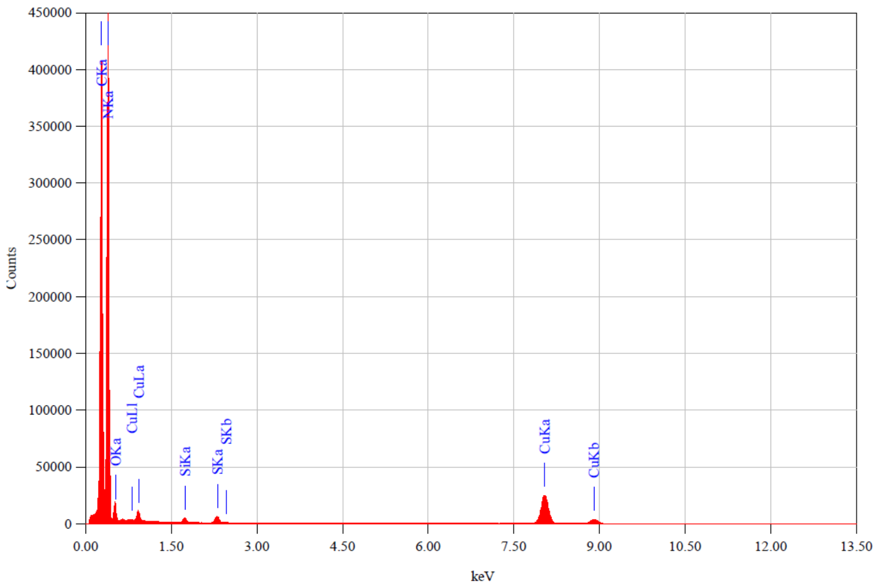

Figure S7. EDS spectra of C_3N_4(P).

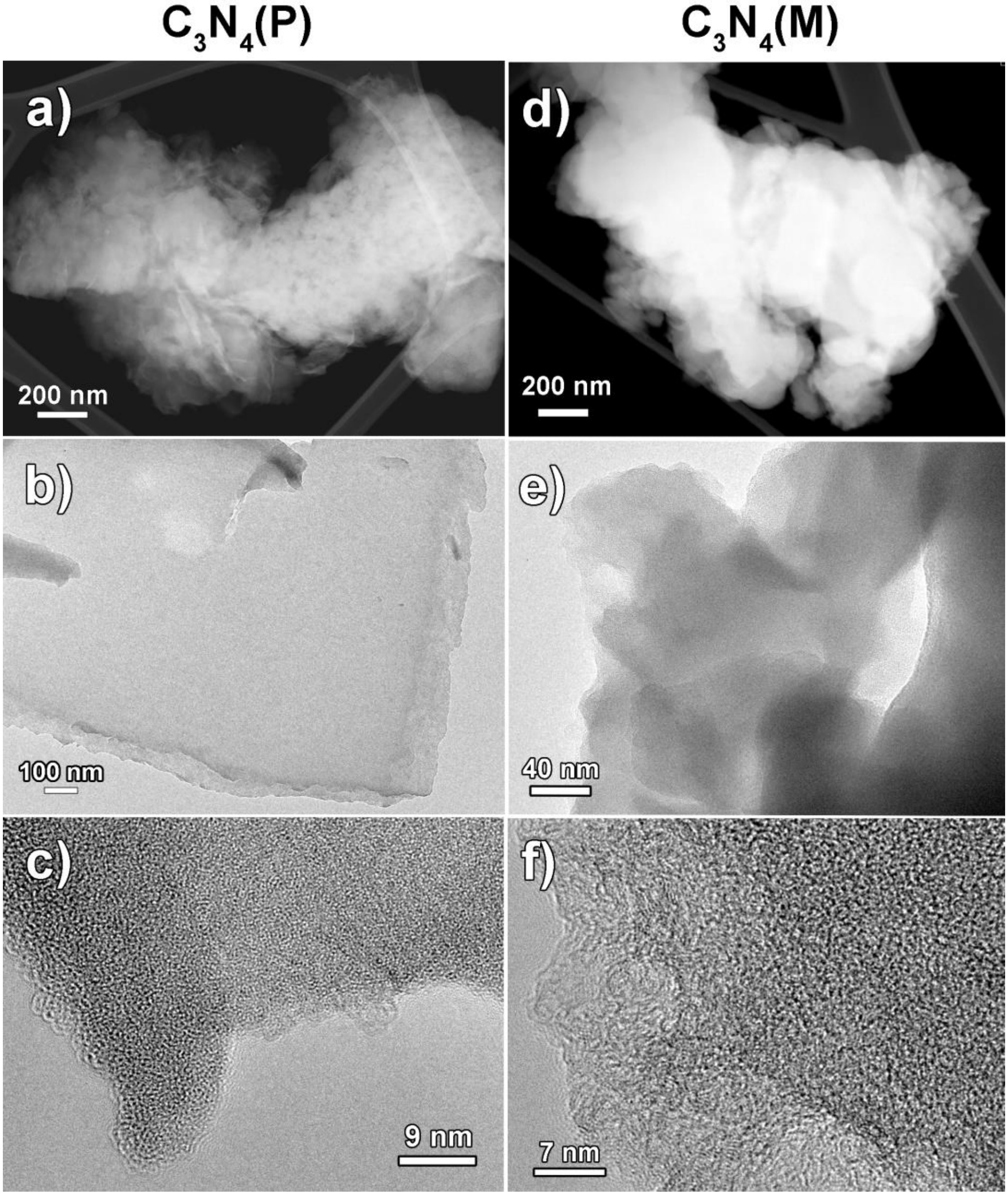

Figure S8. ADF STEM images of a) C_3N_4(P) and d) C_3N_4(M), HRTEM images of b-c) C_3N_4(P) and e-f) C_3N_4(M).

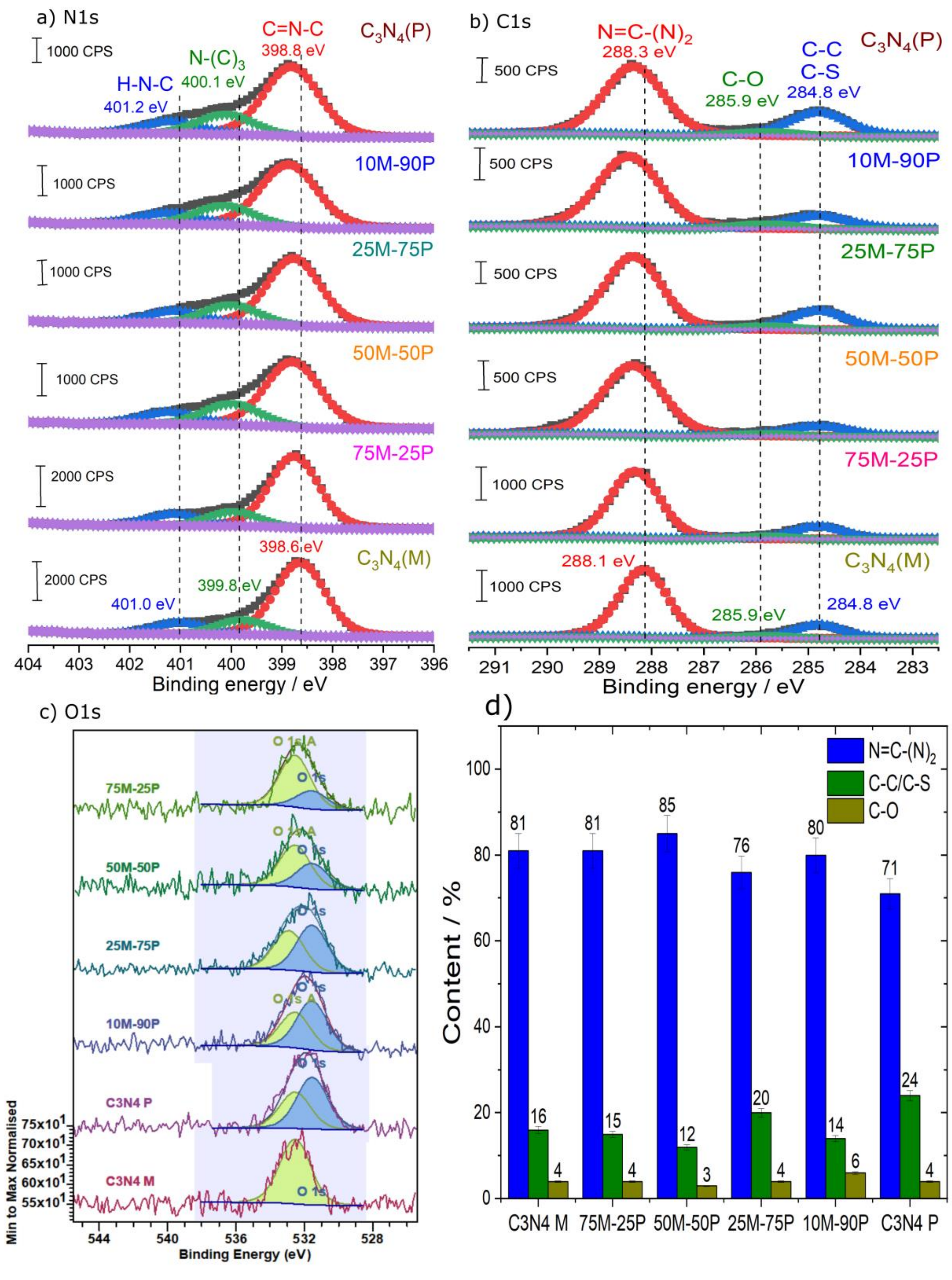

Figure S9. XPS a) N 1s b) C 1s c) O 1s spectra and d) C 1s content distribution of the as-prepared four C_3N_4 hybrids and two C_3N_4 references.

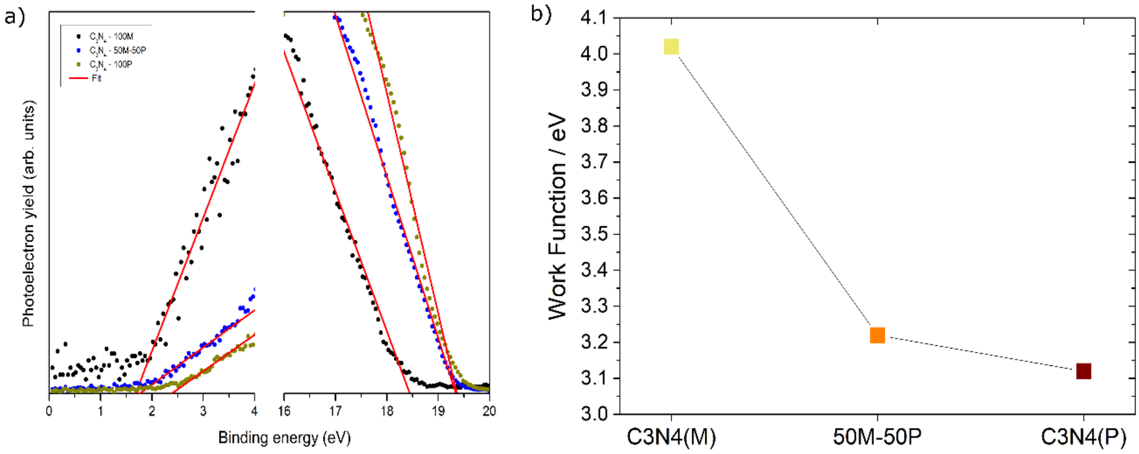

Figure S10. a) UPS spectra b) work function of 50M-50P and 10M-90P hybrids and two C_3N_4 references.

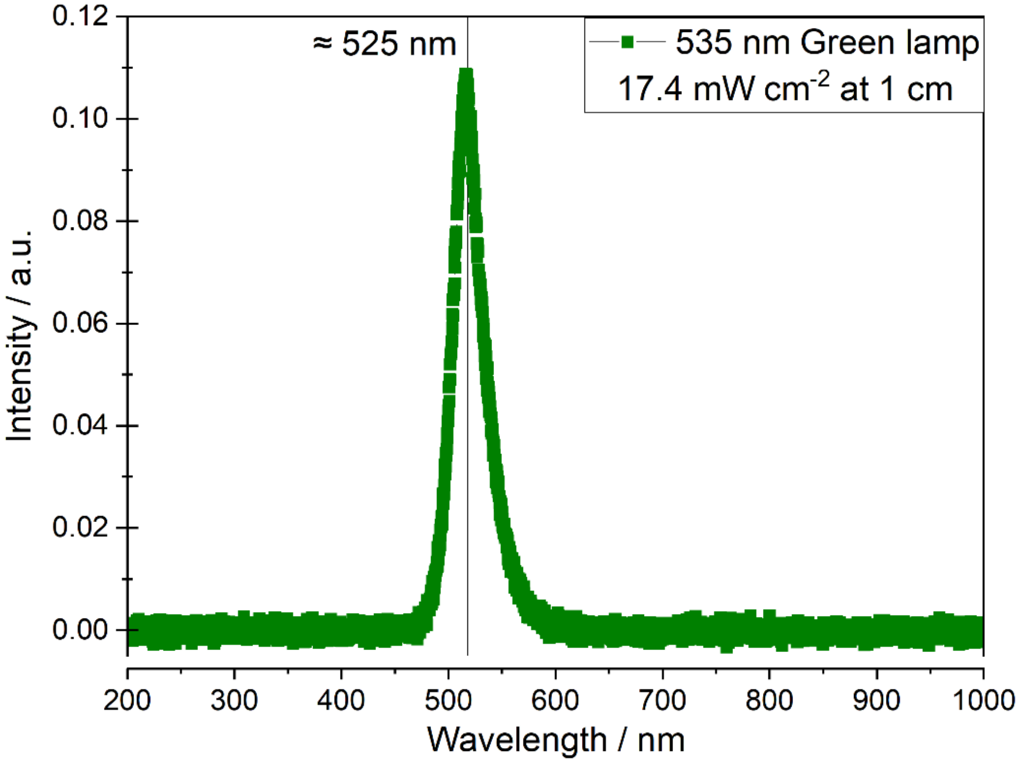

Figure S11. Emission spectra of the LED Green lamp, λ535nm used for photooxidation measurements.

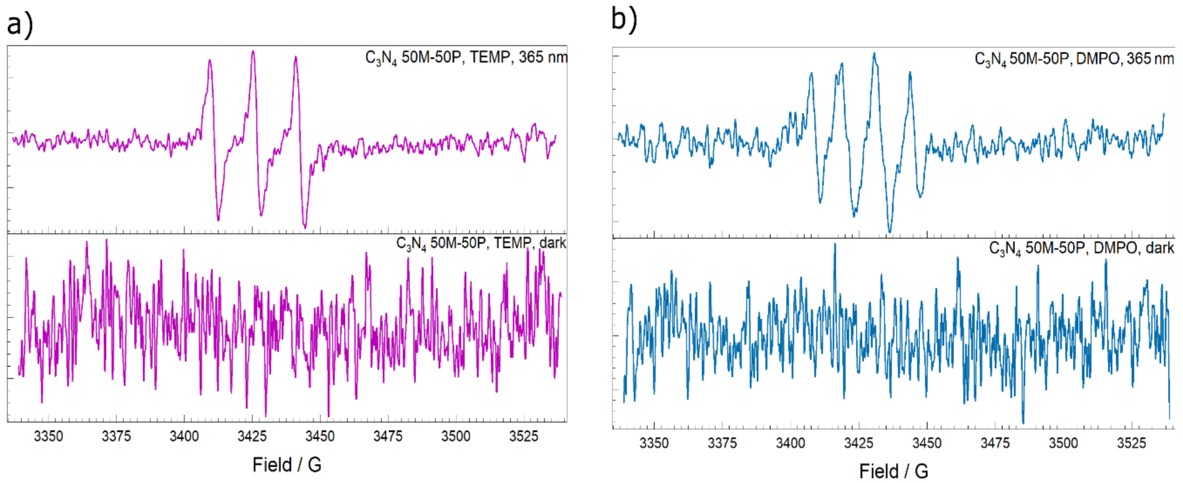

Figure S12. EPR spectra upon 365 nm irradiation and in dark of C_3N_4 (50M-50P) 5 mg and **a)** DMPO **b)** TEMP 30 µL in 1mL MeCN.

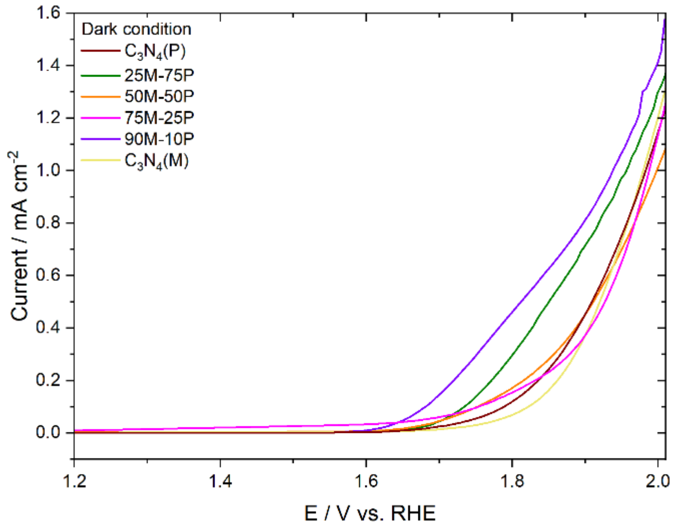

Figure S13. LSV curves in dark conditions of all the as-prepared samples.

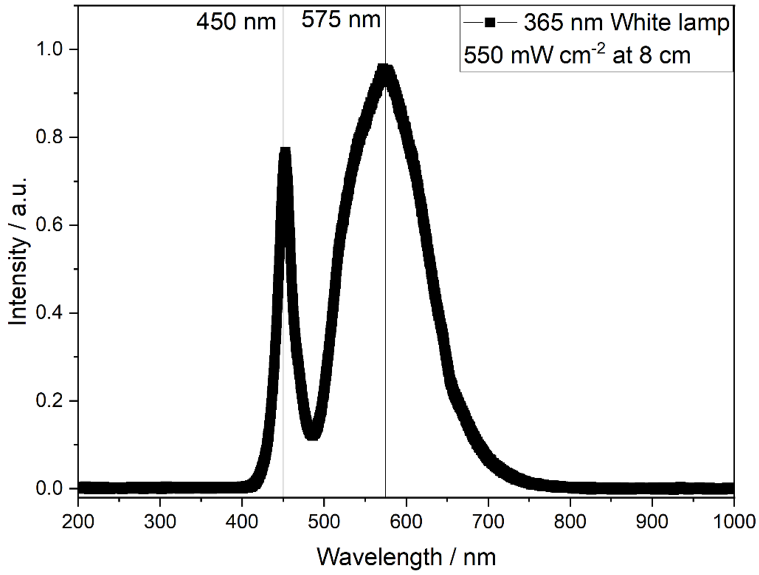

Figure S14. Emission spectra of the LED White lamp, λ=365 nm used for PEC measurements.

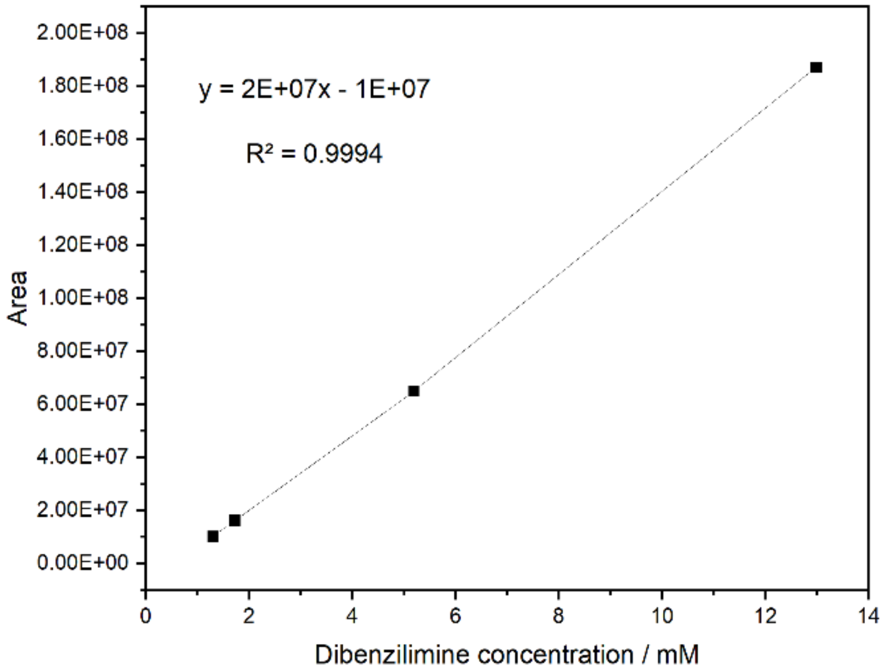

Figure S15. GC calibration curve used for dibenzylimine quantification.

Table S1. Optical and electronic properties of the as-prepared C_3N_4 materials

Sample	BG[Ŧ] / eV	τ[Ɏ] / ns	VB[§] / V vs. NHE	CB[¥] / V vs. NHE
C_3N_4(P)	1.80±0.05	0.09	1.50	-0.30
10M-90P	1.73±0.05	0.15	1.53	-0.20
25M-75P	1.75±0.05	0.62	1.50	-0.25
50M-50P	1.79±0.05	1.07	1.54	-0.25
75M-25P	2.50±0.05	1.49	2.40	-0.10
90M-10P	2.59±0.05	1.64	2.09	-0.50
C_3N_4(M)	2.67±0.05	2.13	2.22	-0.45

Obtained by [Ŧ]=UV-vis-NIR, [Ɏ]=T-PL, [§]= M-S, [¥]= M-S + UV-vis-NIR

Table S2. Elemental analysis of S, C, N, H, and C/N ratio results of the as-prepared C_3N_4 materials

Sample	S	C	N	H	C/N
C_3N_4 (M)	0.1	34.4	62.3	2.0	0.55
90M-10P	0.2	34.9	62.5	1.7	0.56
75M-25P	0.3	34.8	62.1	1.8	0.56
50M-50P	1.6	34.2	61.3	1.9	0.56
25M-75P	1.0	33.7	59.1	1.9	0.57
10M-90P	0.6	33.8	58.9	1.9	0.57
C_3N_4 (P)	2.0	33.4	58.6	1.9	0.57

Table S3. Literature overview of the OER performance indicators of melamine/purpald C_3N_4 hybrids and comparison to reported C_3N_4-based and benchmark catalysts, tested under similar experimental conditions

Material	Onset / V vs. NHE	Tafel slope / mV dec^{-1}	Reference
C_3N_4 (M)	**1.78**	**135**	Our study
C_3N_4 (P)	**1.74**	**160**	
C_3N_4 (90M-10P)	**1.65**	**118**	
C_3N_4 (75M-25P)	**1.66**	**250**	
C_3N_4 (50M-50P)	**1.70**	**182**	
C_3N_4 (25M-75P)	**1.70**	**90**	
PCN/rGO	1.6		1
CoS_x@PCN	1.5		
PCN-CFP (C fiber paper)	1.53	62	2
CN-CFP	1.55	77	
g-C_3N_4/Graphene	1.76	69	3
g-C_3N_4 NS CNT	1.53	83	4
$C_3N_4 – Ti_3C_2$	1.44	75	5
CNTs	1.74	60	6
N-doped carbon	1.6	-	7
P, S-co-doped C_3N_4 sponge	1.26	64	8
S-doped nanostructured C_3N_4	1.42	120	9
Graphitic mesoporous C_3N_4	1.51	52	10
IrO_2	1.49	73	11

References

1. Niu, W. *et al.* Surface-Modified Porous Carbon Nitride Composites as Highly Efficient Electrocatalyst for Zn-Air Batteries. *Adv. Energy Mater.* **8**, 1701642 (2018).
2. Ma, T. Y., Ran, J., Dai, S., Jaroniec, M. & Qiao, S. Z. Phosphorus-Doped Graphitic Carbon Nitrides Grown In Situ on Carbon-Fiber Paper: Flexible and Reversible Oxygen Electrodes. *Angew. Chemie* **127**, 4729–4733 (2015).
3. Tian, J., Liu, Q., Asiri, A. M., Alamry, K. A. & Sun, X. Ultrathin Graphitic C3N4 Nanosheets/Graphene Composites: Efficient Organic Electrocatalyst for Oxygen Evolution Reaction. *ChemSusChem* **7**, 2125–2130 (2014).
4. Ma, T. Y., Dai, S., Jaroniec, M. & Qiao, S. Z. Graphitic Carbon Nitride Nanosheet–Carbon Nanotube Three-Dimensional Porous Composites as High-Performance Oxygen Evolution Electrocatalysts. *Angew. Chemie Int. Ed.* **53**, 7281–7285 (2014).
5. Ma, T. Y., Cao, J. L., Jaroniec, M. & Qiao, S. Z. Interacting Carbon Nitride and Titanium Carbide Nanosheets for High-Performance Oxygen Evolution. *Angew. Chemie Int. Ed.* **55**, 1138–1142 (2016).
6. Cheng, Y. *et al.* Pristine carbon nanotubes as non-metal electrocatalysts for oxygen evolution reaction of water splitting. *Appl. Catal. B Environ.* **163**, 96–104 (2015).
7. Zhao, Y., Nakamura, R., Kamiya, K., Nakanishi, S. & Hashimoto, K. Nitrogen-doped carbon nanomaterials as non-metal electrocatalysts for water oxidation. *Nat. Commun. 2013 41* **4**, 1–7 (2013).
8. Shinde, S. S. *et al.* Scalable 3-D Carbon Nitride Sponge as an Efficient Metal-Free Bifunctional Oxygen Electrocatalyst for Rechargeable Zn-Air Batteries. *ACS Nano* **11**, 347–357 (2017).
9. Kale, V. S. *et al.* Sulfur-Modified Graphitic Carbon Nitride Nanostructures as an Efficient Electrocatalyst for Water Oxidation. *Small* **13**, 1603893 (2017).
10. Wahab, M. A. *et al.* Nanoconfined Synthesis of Nitrogen-Rich Metal-Free Mesoporous Carbon Nitride Electrocatalyst for the Oxygen Evolution Reaction. *ACS Appl. Energy Mater.* **3**, 1439–1447 (2020).
11. Yu, J., Zhong, Y., Zhou, W. & Shao, Z. Facile synthesis of nitrogen-doped carbon nanotubes encapsulating nickel cobalt alloys 3D networks for oxygen evolution reaction in an alkaline solution. *J. Power Sources* **338**, 26–33 (2017).
